# Prevalence and molecular profiling of Epstein Barr virus (EBV) among healthy blood donors from different nationalities in Qatar

**DOI:** 10.1371/journal.pone.0189033

**Published:** 2017-12-11

**Authors:** Maria K. Smatti, Hadi M. Yassine, Raed AbuOdeh, Asmaa AlMarawani, Sara A. Taleb, Asmaa A. Althani, Gheyath K. Nasrallah

**Affiliations:** 1 Biomedical Research Center, Qatar University, Doha, Qatar; 2 Department of Biomedical Science, College of Health Sciences, Qatar University, Doha, Qatar; 3 Department of Medical Laboratory Sciences, College of Health Sciences, University of Sharjah, Sharjah, U.A.E; 4 Department of Laboratory, Aspetar Orthopaedic and Sports Medicine Hospital, Doha, Qatar; University of North Carolina at Chapel Hill, UNITED STATES

## Abstract

**Background:**

The Epstein–Barr virus (EBV) is the causative agent of infectious mononucleosis. EBV is highly prevalent lymphotropic herpesvirus and has been linked to several malignancies. Transmission is generally by oral secretions, but can be through blood transfusions and organ transplantations. This study aimed to determine the seroprevalence, viremia rates, and circulating genotypes of EBV in healthy blood donors in Qatar.

**Methods:**

Blood samples from 673 blood donors of different nationalities residing in Qatar (mainly Qatar, Egypt, Syria, Jordan, Pakistan, and India) were collected and tested for anti-EBV capsid (VCA; IgG & IgM), nuclear (EBNA; IgG), and early (EA-D; IgG) antigens. Avidity testing was determined when active infection was suspected. DNA was extracted from the buffy coat and subjected to EBV-DNA quantification using qRT-PCR. Genotyping was performed using nested-PCR targeting EBV-EBNA2 gene, and phylogeny by sequence analysis of the LMP-1 gene.

**Results:**

97.9% (673/659) of the samples were seropositive as indicated by the presence VCA-IgG, while 52.6% (354/673) had detectible EBV-DNA. EBV seroprevalence and viremia rates increased significantly with age. Genotyping of 51 randomly selected samples showed predominance of Genotype 1 (72.5%, 37/51) as compared to genotype 2 (3.5%), and mixed infections were detected in 4% of the samples. Sub-genotyping for these samples revealed that the Mediterranean strain was predominant (65.3%), followed by B95.8 prototype and North Carolina strains (12.2% each), and China1 strain (6%).

**Conclusion:**

As a first study to evaluate EBV infection in highly diverse population in Qatar, where expatriates represent more than 85% of the population, our results indicated high seroprevalence and viremia rate of EBV in different nationalities, with genotype 1 and Mediterranean strain being predominant. Clinical significance of these finding have not been investigated and shall be evaluated in future studies.

## Introduction

Epstein Barr virus (EBV), or human herpesvirus 4, is a lymphotropic herpesvirus and the causative agent of infectious mononucleosis. Although the virus was first discovered in cells isolated from African Burkitt’s lymphoma, further studies revealed its high prevalence worldwide [1]. Like other herpesviruses, EBV causes latent infection. It primarily infects epithelial cells and spreads to B lymphocytes where it persists for life [2]. In normal hosts, B lymphocytes and epithelial cells are the cellular targets for EBV primary infection. However, EBV can infect a wide range of non-B lymphocytes which determine the development and pathogenesis of EBV related diseases [3]. It is estimated that more than 90% of the world’s population is EBV-seropositive [4]. Typically, primary infection with EBV occurs in childhood resulting in mild or no disease, however, adulthood infection with the virus may lead to infectious mononucleosis (IM) [5]. Further, this virus has been linked to a wide range of diseases including multiple sclerosis (MS), and malignancies, such as gastric carcinoma, Hodgkin’s lymphoma and nasopharyngeal carcinoma (NPC) [4–7].

EBV encompasses a 172 kbp double stranded DNA genome that codes for more than 85 genes [5, 6]. In latency, only small subset of viral genes are expressed, which include the six EBV nuclear proteins: EBNA-1, EBNA-2, EBNA-3A, EBNA-3B, EBNA-3C, EBNA-LP, and three latent membrane proteins: LMP-1, LMP-2A, LMP-2B [5].

There are two main EBV genotypes, Type 1 and Type 2, or Type A and B, distinguished by the differences in the EBNA-2 gene, which exhibits only 54% homology between the two types [8]. EBV types 1 and 2 can further be subdivided into different virus strains based on the genetic diversity of LMP-1gene, which shows greater degree of polymorphism than most EBV genes [9]. LMP-1 is a 356-amino acid protein, which consists of a short cytoplasmic N-terminus, six membrane spanning domains, and a long cytoplasmic C- terminal domain [10]. LMP-1 plays an important role in signal transduction and cell survival [6]. Variants in LMP-1 were classified into 7 main groups: B95-8, Alaskan, China 1, China 2, Med+, Med- and NC [4, 6, 11]. However, new strains were subsequently reported from different origins, including two new strains from Thailand, Southeastern Asia 1 (SEA1), and Southeastern Asia 2 (SEA2), which have unique amino acid substitutions [12, 13]. Multiple EBV variants could be detected within one individual, which could affect disease induction and prognosis [14]. For example, a variant LMP-1 gene with 30 bp deletion gene was detected in virus isolated from NPC tumor and was associated with a higher transforming activity compared to the typical prototype LMP-1 (B95-8) [15, 16]

EBV is primarily transmitted through the oral route; however, it has been reported that blood transfusion and organ transplantation are also other feasible transmission routes of EBV [17–19]. Although blood-banking organizations adopt strict regulations to minimize the risk of transfusion transmission of pathogens, nonetheless, there are still concerns regarding transmission of untested pathogens, such as EBV [20]. Blood banks rely on the leukoreduction process to ensure the safety of blood products. Although leukoreduction procedure can pointedly reduce the number of EBV genome, it has been found that EBV can still be detected in leukoreduced products [21]. In conclusion, leukoreduction doesn’t completely eliminate cells harboring EBV, which might poses a potential risk for blood products recipients, especially in the immunocompromised [20, 21].

The lack of a licensed protective vaccine or specific treatment against EBV increases the risk of EBV spread. The vast majority of published studies on EBV prevalence are focused on serological analysis rather than viremia detection [22–26]. Clearly, detection of circulating EBV DNA is a better indication of infection status, which can contribute to improving the level of medical care prevention measures [27, 28]. Only few studies have looked at EBV viremia in healthy subjects by detecting blood DNA [29–31]. Previous studies from Middle Eastern countries such as Jordan [32], Kuwait [33], Egypt [34], Saudi Arabia [35], the UAE [36], and Syria [37] have investigated EBV and its association with certain diseases such as Hodgkin’s lymphoma (ranging from 28% to 87%), but not among healthy individuals. To the best of our knowledge, no studies have been conducted in Qatar or the region concerning EBV detection and genotyping in neither cancer patients nor healthy donors. Importantly, population in Qatar is multinational with expatriates constituting more than 85% [38]. This study aimed at estimating the EBV infection rates among healthy blood donors in Qatar, and determining the demographic distribution of genotypes and sub-genotypes in relation to gender, age, and ethnicity. This information will enable the health officials in Qatar to consider the development of new policies that aim at reducing the burden of communicable diseases related to blood transfusion.

## Materials and methods

### Sample collection and ethical approval

A total of 673 whole blood samples (223 from Qataris and 450 from other nationalities) were collected in EDTA tubes from healthy donors at the Blood Donor Unit at Hamad Medical Corporation (HMC) over a period of one year (September 2014 –September 2015). Blood samples were handled and stored following standard safety procedures and guidelines. This study was approved by HMC-Institutional Review Board (HMC-IRB #14292/14) and Qatar University IRB (QU-IRB 518-EA/15). See ethical compliance section below.

#### Ethical compliance

All procedures performed in studies involving human participants were in accordance with the ethical standards of the Hamad Medical Corporation (HMC) and Qatar University (QU). HMC-Institutional Review Board (HMC-IRB #14292/14) and QU-IRB (#518-EA/15) were obtained before sample collection. This research involved no risk to the subjects. Their rights and welfare was not affected. That is, samples were anonymously collected and accompanied without names or sensitive information related to the patients. Thus, a waiver of all the consent requirements was obtained before starting sample collection from both QU and the HMC IRB committee. Only general information (age, sex, and nationality) was collected and kept confidential.

### Anti-EBV serology testing

#### Qualitative ELISA testing of anti- EBV antibodies

To determine EBV seroprevalence, commercial ELISA kits (Diagnostic Automation, USA) were used to screen plasma samples for the presence of EBV antibodies. A panel test was performed; this included screening antibodies for: viral capsid antigen (VCA) [both VCA- IgG (Catalog # 1405–2), and VCA- IgM (Catalog # 140692)], Epstein Barr nuclear antigen -1 (EBNA-1) -IgG (Catalog # 1425–1), and EBV early antigen (EA)-IgG (Catalog # 1415–11). All ELISA assays were based on the same principle. In each kit, purified EBV antigens were coated on the surface of polystyrene microwells to bind to the specific complementary antibodies. Kit controls were included in each assay. For each assay, the cutoff calibrator value was calculated by multiplying the correction factor which is specific for each kit, by the mean calibrator OD. The immune status ratio (IRS) was calculated by dividing the samples OD value by the cutoff calibrator value. Samples were considered positive if the IRS value was above the IRS cut-off value. Samples were retested when the IRS value was in the equivocal range. According to the serological patterns obtained from the ELISA screening of the four antibodies (VCA-IgG and IgM, EBNA-IgG, EA-IgG), samples were classified into four stages (No infection, active infection, past infection, and reactivation [39, 40]) according to the following criteria: 1. The presence of VCA-IgG and VCA-IgM and EA-IgG in the absence of EBNA-IgG indicates acute primary infection; 2. Presence of VCA-IgG only, or VCA-IgG and EBNA-IgG without VCA-IgM and EA-IgG indicates past infection; and 3. Presence of VCA-IgG, EBNA-IgG and EA-IgG in the absence of VCA-IgM indicates possible viral reactivation [41]. Samples yielding uncertain diagnosis such as the positivity of all antibodies, or the presence of VCA-IgM and IgG with EBNA-IgG were further investigated by VCA-IgG avidity assay to obtain a definitive diagnosis. Table 1 summarizes the interpretation of EBV serological patterns.

**Table 1:** Interpretation of EBV serological pattens in immunocompetent individuals ^a^.

Interpretation	Anti- EBV antibodies			
	VCA- IgM	VCA-IgG	EBNA-IgG	EA-IgG
No infection	-	-	-	-
Acute infection	+	+	-	+
Past infection	-	+	+	-
Reactivation	-	+	+	+

^a^ Summarized from References [39–41].

Information related to patients, serology and PCR results are presented in S1 Table.

#### IgG avidity confirmatory test

VCA IgG avidity assay was performed when the ELISA results produced a doubtful diagnosis, or when active infection was suspected. EBV capsid antigen (EBV-CA) avidity determination kit (Cat # EI 2791-9601-1 G, Euroimmun, Germany) was used. Briefly, two microplate ELISA tests were performed at the same time. One plate was used for conventional ELISA procedure, and the other plate was treated with 8 M urea as a dissociating agent to separate low avidity antibodies. Following the manufacturer’s instructions, urea was added after the incubation period with patient’s serum, and before adding the secondary anti-human IgG. The difference in the reading with or without urea treatment in both plates was evaluated by photometric measurement at wavelength of 450 nm. Moreover, for a reliable interpretation, the relative avidity index (RAI) was calculated to compare between values with and without urea; RAI = the value with urea / value without urea. High RAI values indicated past infection, while low RAI values indicated acute infection [42].

### PMNCs separation

Ficoll-Paque PLUS (GE Healthcare Life Sciences, USA) was used for the isolation of lymphocytes from blood samples (n = 673) collected in EDTA tubes as per manufacturer’s instructions. Briefly, 1X Hank’s balanced salt solution (Life Technologies, USA) was used for 1:1 dilution of the blood samples and the diluted blood was layered over the Ficoll-Paque PLUS solution. After centrifugation at 400× g for 30–40 minutes at 20°C, blood cells differentially migrated, resulting in the formation of different layers containing different cell types. The layer in the bottom contained aggregated erythrocytes, and the buffy coat containing polymorphonuclear cells (PMNCs), including lymphocytes, were at the interface between Ficoll reagent and the plasma. Plasma was discarded and PMNCs were collected in a separate tube. Finally, PMNCs were subjected to short washing steps using a balanced salt solution to remove any platelets, Ficoll-Paque PLUS and remaining plasma. Isolated PMNC were stored at -20°C in PBS until performing DNA extraction.

### DNA extraction

DNA was extracted from PMNCs (buffy coat samples) suspended in 200 μl of PBS using Qiagen kit following the manufacturer’s instructions (Catalog # 51106, Qiagen, Germany). The concentration and the purity of all extracted DNA samples were measured using NanoQuant microplate reader (Infinite pro200, Tecan, Switzerland). Extracted DNA samples were then stored at -20°C for further testing.

### EBV DNA detection by real time PCR

Detection and copy number quantification of EBV DNA in all extracted samples (n = 673) were performed using a real-time PCR detection and quantification kit (Catalog # V48-100FRT, Sacace, Italy) using 10 μl of DNA as per manufacturer’s instructions. The principle of the detection in this kit is based on using real time amplification with fluorescent reporter dye probes specific for EBV LMP gene. This kit also contained an indigenous internal control (IC) that amplifies β-globin gene. QuantStudio™ 6 Flex Real-Time PCR reader (Applied Biosystems, USA) was used for detection of the fluorescent dyes. Calibrators were used to construct the standard curve that was used to quantify EBV copies in the tested samples. The reaction was considered valid only if the quantity of IC was more than 2000 copies per reaction, and no amplification was detected in the negative control. EBV viral load was calculated in copies per reaction, copies per one microgram of extracted DNA, and copies per one ml of blood.

### EBV genotyping by nested PCR of the EBNA-2 gene

EBV genotyping was performed using nested PCR targeting the EBNA2 gene as previously described [43, 44]. Briefly, in the first round of amplification, primers E2p1 (5’-AGGGATGCCTGGACACAAGA-3’) and E2p2 (5’-TGGTGCTGCTGGTGGTGGCAA T-3’) were used to amplify a fragment of 596 bp covering almost the entire EBNA2 gene. In the second round of amplification, primers Ap1 (5’- TCTTGATAGGGATCCGCTAGGATA-3’) and Ap2 (5’-ACCGTGGTTCTGGACTATCTGGATC-3’), were used to amplify a 497 bp fragment which identifies the EBV type-1 EBNA2 gene product, whereas primers Bp1 (5’-CATGGTAGCCTTAGGACATA-3’) and Bp2 (5’-AGACTTAGTTGATGCCCTAG-3’) amplified a 150 bp fragment that characterizes EBV type-2 EBNA2 gene product. Samples with mixed EBV infections were characterized by the presence of two amplicons, 479 bp in length using AP primers, and 150 bp using BP primers. PCR reaction of 50 μl was prepared in 0.2 ml microcentrifuge tube using HotStarTaq DNA Polymerase kit (Catalog # 203203, Qiagen, Germany). Reaction mix contained 0.25 μl of HotStarTaq DNA Polymerase, 10 μl of 5x Q-Solution, 5 μl of 10x PCR buffer which already contained 15mM MgCl_2_, and 1 μl of 10mM dNTP's (Catalog # N0447S, New England Biolabs, USA). 1 μl of 10μM/ μl of one set of the above primers were added to the mixture. For the first round of PCR using E2p1 and E2p2 primers, amplification conditions were as follows: after an initial heat activation step of 15 min at 95°C, 40 cycles of amplification were performed: denaturation for 5 min. at 95°C, annealing for 1 min. at 58°C, and extension for 1 min. at 72°C, followed by a final extension step of 10 min. at 72°C. For the second round of PCR, the same amplification conditions were used except for the annealing temperature: 63°C was used for Ap1 and Ap2 primers, and 53°C was used for Bp1 and Bp2 primers. PCR amplified products were separated on 2% agarose and visualized using UV gel documentation system (Bio-Rad, US). In all experiments, a negative control (sterile water instead of DNA) and a positive control were used.

### EBV sub-genotyping by sequencing of LMP-1 gene

#### Nested PCR of LMP-1 gene

EBNA-2 genotyped samples were further sub-genotyped targeting LMP-1 gene, which has a high degree of polymorphism among EBV genes [4]. A nested PCR of the LMP-1 gene was done as previously described [9], in the first round of amplification, primers A1 (5’-AGTCATAGTAGCTTAGCTGAA-3’) and A2 (5’-CCATGGACAACGACACAGT -3’) were used to amplify a fragment of 602 bp covering the C-terminus of LMP-1 gene In the second round of amplification, primers B1 (5’-AGTCATAGTAGCTTAGCTGAA-3’) and B2 (5’- CAGTGATGAACACCACCACG-3’) amplified a 587 bp fragment [9]. PCR reactions were prepared similar to the EBNA-2 amplification procedure and using the same kit and reagents, but with the designated primers. Amplification conditions were as follows: initial heat activation step of 15 min at 95°C; 40 cycles of PCR amplifications: 5 min. at 95°C, 1 min. at 53°C and 1 min. at 72°C; and a final extension step of 10 min. at 72°C. Amplified products were analyzed by 2% agarose gel electrophoresis and visualized using UV gel documentation system. In each experiment, a negative control (sterile water instead of DNA) and positive control were used.

### Cloning

LMP-1 PCR products from 51 randomly selected samples were cloned, sequenced and compared to sequences deposited in NCBI GenBank followed by phylogenetic analysis. To do that, PCR products (587 bp) were purified using PCR purification kit (Qiagen; Germeny), and cloned by TA-cloning into pDrive vector using Qiagen cloning kit following the manufacturers' instructions. As previously described by AbuOdeh et al [45], three μl of the ligated plasmids were transformed by electroporation into 50 μL *E*. *coli* DH5α electrocompetent cells (Invitrogen, USA). Transformed cell were then allowed to recover using 300 μl SOC broth (Invitrogen) and incubated with shaking for 30 min at 37°C. The 300 μl of SOC bacterial suspensions were then spread on LB Agar containing 100 μg/ml ampicillin (Sigma, USA). Following an overnight incubation, several distinct colonies from each culture plate were randomly picked, suspended in LB broth containing 100 μg/ml ampicillin and grown with shaking for 24 hrs. Plasmids were purified from cultured bacteria using a QIAprep® Spin Miniprep Kit (Catalog # 231122, Qiagen, Germeny). Prior to sequencing, amplicons were examined for the presence of the cloned PCR products using restriction digestion with *Eco*RI enzyme. Digested samples were then analyzed in 2% agarose gel electrophoresis. Plasmids harboring the cloned fragment yielded a 587 bp band and a thicker large plasmid band. Samples were subsequently sequenced at MC lab (California State, U.S.) using ABI 3730XL sequencer, using T7 forward primer and Sp6 reverse primer.

### Phylogenetic analysis

LMP-1 gene nucleotide sequences were translated to amino acid sequences and CLC sequence viewer (version 7.1.1. Aarhus, Denmark) was used to run sequence alignments and construct the phylogenetic tree. For sequence homology comparison, sequences were compared to reference sequences representing the seven main EBV strains available in the GenBank database: B95.8 prototype strain (V01555), Med + with 30-bp deletion (AY337721), Med -without 30-bp deletion (AY493810), China 1 (AY337723), China 2 (AY337724), Alaskan (AY337725), and NC strain (AY337726). As previously described by Saechan et al [13], sequences were aligned using Clustal W method, and the phylogenetic tree was generated using the neighbor joining method. Bootstrapping and reconstruction were carried out with 1000 replicates to obtain the confidence level of the phylogenetic tree.

### Statistical analysis

To determine the relation significance between variable ratios, chi-square test was used. For the correlation between EBV serology and real time PCR results, Kruskal Wallis test and Mann–Whitney *U* test were used. Results with *p*-value < 0.05 were considered statistically significant. The statistics software SPSS.23 and GraphPad Prism 7.00 were used for data analysis.

## Results

### Seroprevalence of EBV among healthy blood donors in Qatar

Table 2 summarized the demography of the studied population. A total of 673 blood samples were analyzed in the present study, of which, 659 samples (97.9%) were from males and 14 samples (2.1%) from females. The majority of the samples were obtained from non-Qatari residents (66.9%), and the rest were from Qatari individuals (33.1%). The age of participants ranged between 19 and 68 years, (37.12 ± 9.3 years).

**Table 2:** Demographic characteristics of the studied subjects (Total N = 673).

Category		No. (%)
Gender		
	Male	659 (97.9)
	Female	14 (2.1)
Nationality		
	Qatari	223 (33.1)
	Male	219 (98.2)
	Female	4 (1.8)
	Non-Qatari	450 (66.9)
	Male	440 (97.8)
	Female	10 (2.2)
Age		
	<20–30	176 (26.15)
	31–40	264 (39.2)
	41–50	174 (25.9)
	>50	59 (8.8)

Initially, all plasma samples (673) were screened for the presence of anti-EBV antibodies (VCA- IgG, VCA- IgM, EBNA1-IgG, EA-IgG) using ELISA. 97.9% (659/673) of the samples were seropositive for at least one of the aforementioned anti-EBV antibodies. A seroprevalence of 91.5% (n = 616) was detected for anti-EBNA IgG antibodies. High percentage of samples 97.9% (n = 659) were positive for VCA- IgG, while only 1.8% (n = 12) were positive for IgM class antibodies targeting the same gene. 10.6% (n = 71) samples tested positive for EA-IgG antibodies.

VCA IgG avidity assay was then performed as it distinguishes between past and active infection. The strength of IgG binding was evaluated in all samples that showed ambiguous serological interpretation and for samples with suspected active infection (n = 15). All tested samples (n = 15) had high RAI, which indicates the presence of high avidity mature IgG antibodies, thus, excludes the possibility of an active infection. Accordingly, final serological analysis indicated that the majority of individuals (594 out of 679; 88.26%) had a past infection with the virus, while only 9.7% (n = 65) had viral reactivation. Further, there was no sign of past EBV infection in the remaining 2.1% (n = 14) samples (Table 3).

**Table 3:** EBV seroprevalence and viremia rates in the studied population (N = 673).

Category		Total No.	EBV serology			EBV DNA		
			Positive		*p*-value*	Positive		*p*-value*
			No. (%)			No. (%)		
Gender								
	Male	659	645 (97.9)		0.581	347 (52.7)		0.467
	Female	14	14 (100)			6 (42.9)		
								
Nationality								
	Qatari	223	218 (97.8)		0.8358	123 (55.2)		0.296
	Male	219	214 (97.7)			112 (51.1)		
	Female	4	4 (100)			2 (50)		
	Non-Qatari	450	441 (98)			229 (50.9)		
	Male	440	431 (97.9)			226 (51.4)		
	Female	10	10 (100)			4 (40)		
Age Group								
	<20–30	176	169 (96)		0.037	79(44.8)		0.009
	31–40	264	257 (97.3)			134(50.76)		
	41–50	174	174 (100)			100 (57.5)		
	> 51	59	59 (100)			40(67)		

* Pearson Chi^2^ p-value

### Association of EBV seroprevalence with age, gender, and nationality

Association between EBV seroprevalence and gender, age, and geographic origin of the participants was performed using chi-square test. There was no statistically significant association (*p* = 0.58) between EBV seroprevalence and gender, male (97.7%) and females (100%), probably because of the difference in samples size between the two groups.

Since Qatar is a multinational country where expatriates constitute more than 85% of the population [38], we investigated EBV frequency between Qataris (n = 223) and non-Qatari (n = 450) residents, specifically, among six major nationalities: Syria (n = 95), Egypt (n = 92), Jordan/Palestine (n = 61), India (n = 59), and Pakistan (n = 20). We found no significant difference in EBV seroprevalence between Qataris and non- Qataris (*p* = 0.84), where 218 out of 233 (97.8%) Qatari donors were seropositive compared to 441 out of 450 (98%) seropositive donors from other nationalities. Egyptians and Pakistanis had the highest rates of EBV seroprevalence (100%) followed by Indians (98.3%), Syrians (96.8%), Qataris (97.8), and Jordanian/Palestinian nationalities (95.1%). There was no statistical significance in EBV seroprevalence nor the stage of infection among the different nationalities (p = 0.99) (Table 4)

**Table 4:** EBV serology and viremia rates among major nationalities (N = 673).

Category	Total No.	Seropositive o. (%)	Positive RT-PCR No. (%)	p-value*
Nationality				
Qatar	223	218 (97.8)	123 (55.2)	0.99
Syria	95	92 (96.8)	51 (53.7)	
Egypt	92	92 (100)	47 (51.1)	
India	59	58 (98.3)	28 (47.5)	
Jordan/Palestine	61	58 (95.1)	28 (45.9)	
Pakistan	20	20 (100)	13 (65)	
Others	123	121 (98.4)	64 (52)	
Total	673			

* Pearson Chi^2^ p-value

**Fig 1:**
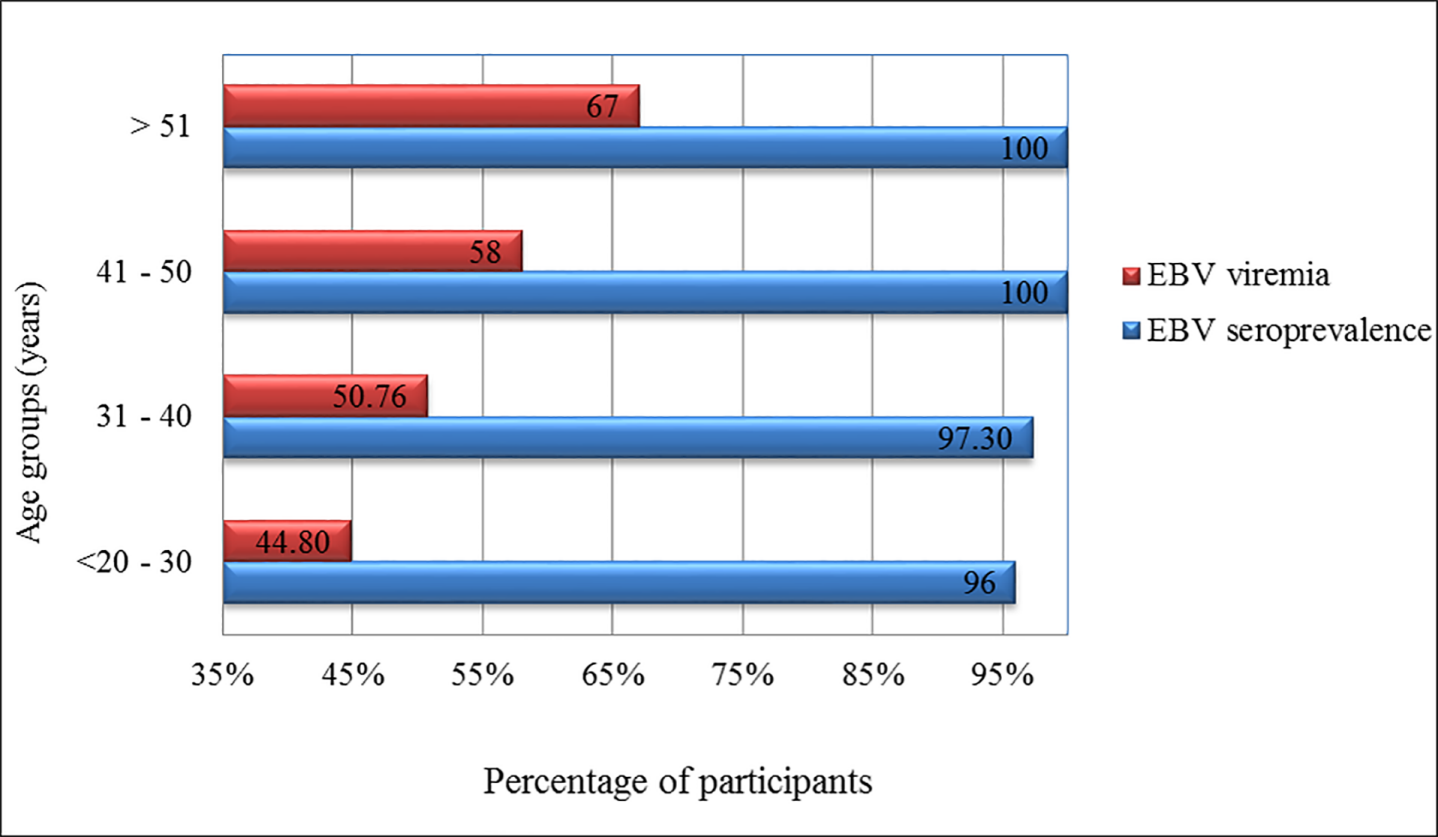
The correlation of EBV seroprevalence and viremia rates with the ages of donors. **Both seroprevalence and viremia rates increased significantly with age**.

We then investigated the correlation of EBV seroconversion and age of donors and expectedly, infection rates increased significantly with age (*p* = 0.03), from 96% seropositivity in individuals less than 30 years old to 100% in people above 40 years of age (Fig 1).

### EBV viremia rates among healthy blood donors

Viral load was determined in 673 blood samples using quantitative real-time (qRT-PCR) targeting LMP-1 gene. EBV DNA was detected in 52.6% (n = 354) samples. There was no significant correlation between viremia rates and gender (males (52.8%) and females (42.9%)), or nationality (Qataris (55.2%) and non-Qataris (51.1%)). However, there was a direct correlation between viremia rates and age (*p*-value = 0.009) among tested donors. The lowest viremia rate was detected in age group less than 30 years (44.8%) compared to donors above 51 years of age (67%) (Table 3, Fig 1). Viral load in the positive samples ranged between 0.915–2585.5 copies/ml of blood, with a mean of 68.23 copies/ml and SD of 183.5.

### Correlation between serology and viremia rates

Correlation between ELISA serological data and EBV DNA in blood was investigated. All EBV seronegative donors had undetectable EBV DNA in their blood (n = 14). 53% (315/594) of individuals with past infection status had detectable levels of EBV DNA in their blood. Further, 60% (39/65) of the samples from individuals with reactivation stage were PCR positive for EBV (Table 5).

**Table 5:** Correlation between EBV serology and RT-PCR.

Category by ELISA	Total No.	EBV DNA detected by RT-PCR			
		Positive No. (%)	Negative No. (%)	Viral load copies/ml of blood	Viral load copies/μg of DNA
No infection	14	0 (0)	14 (100)	0	0
Past infection	594	315 (53)	279 (47)	0.915–2585.5	18.3–51,710
Reactivation	65	39 (60)	26 (40)	2.5–1026.8	50–20,586
Total	673	354	319	0.915–2585.5	18.3–51,710

### Circulating EBV genotypes and sub-genotypes among blood donors

**Fig 2:**
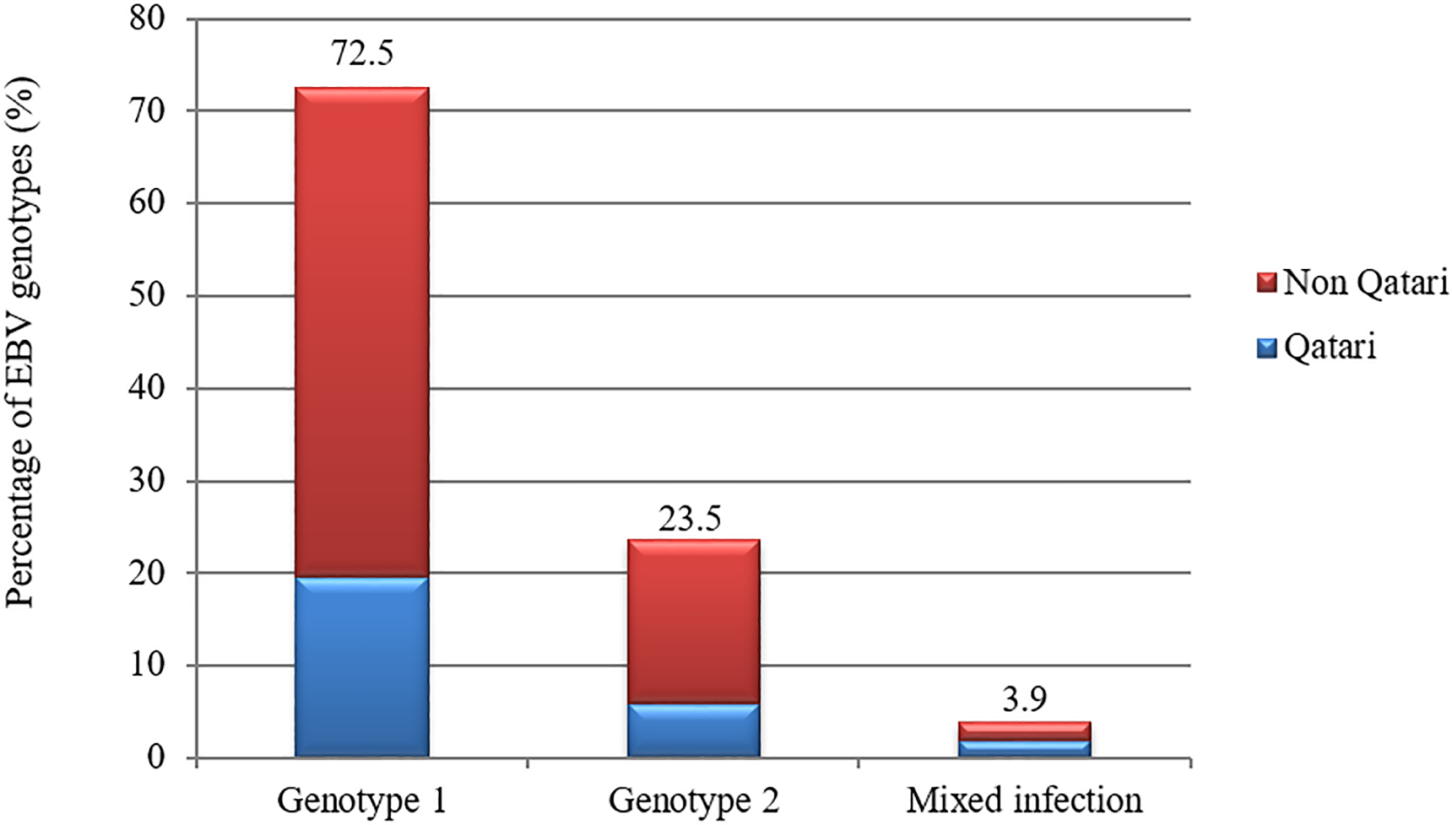
The distribution of EBV genotypes among Qatari and non- Qatari residents.

51 samples from different nationalities were randomly selected for genotyping targeting EBNA2 gene. EBV type 1, which is common in Europe, America, China, and South Asia [4, 15] was predominant (72.5%) in both Qatari and non-Qatari populations. On the other hand, 23.5% (n = 12) of the samples were positive for type 2 and 3.9% (n = 2) of the samples had mixed infections (Fig 2, S1–S3 Figs).

**Fig 3:**
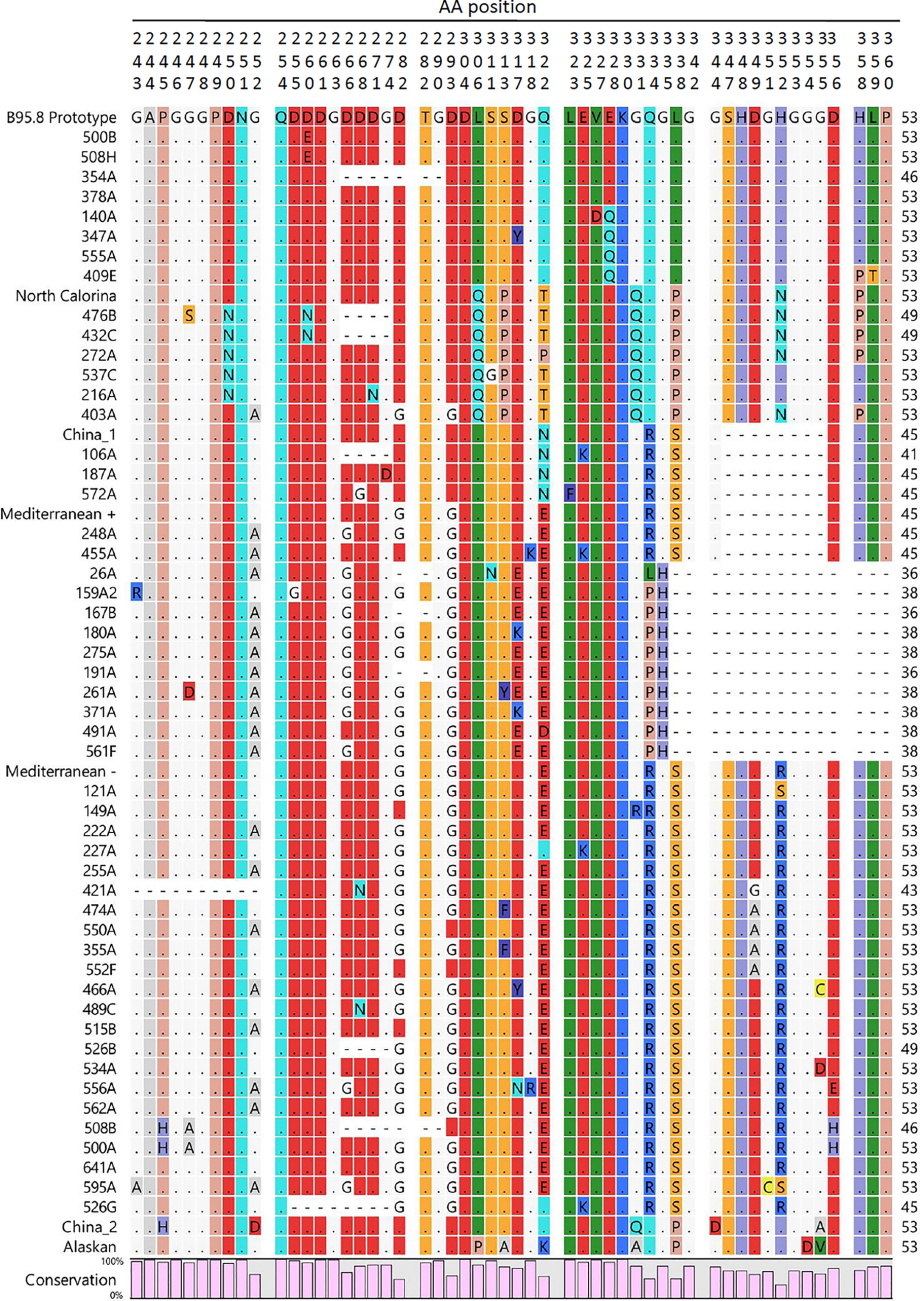
Alignment of the LMP-1 C-terminal amino acid sequences by Clustal W method using CLC 7.7.1 sequence viewer software. Numbers on the top represent the amino acid position corresponding to the prototype (B95.8). Dots indicate identical amino acid to the prototype sequence, while dashes indicate deletions.

**Fig 4:**
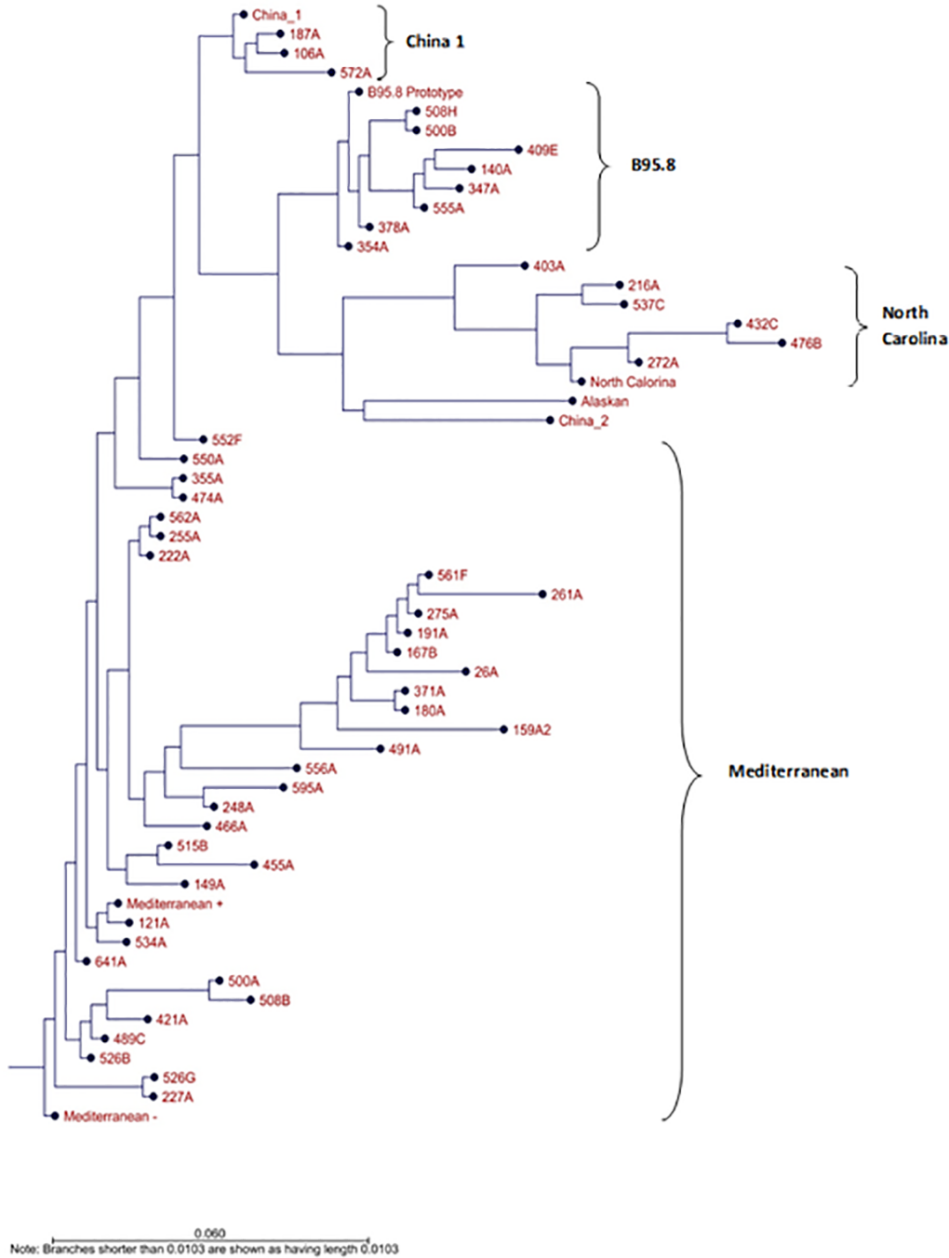
Phylogenetic tree of LMP-1 C-terminus amino acid sequences using neighbor-joining method and Kimura two-parameter algorithm.

The 51 genotyped samples were further sub-genotyped by sequence analysis of the LMP-1 gene C-terminus region. Nested PCR was used to amplify a 587 bp product that was subsequently cloned into pDrived plasmid (S4 and S5 Figs). Three to six clones from each sample (total of 200 clones) were sequenced using ABI 3730XL sequencer (Applied Biosystems, USA). Generated sequences (coordinates 168160 to 168748) were translated to amino acid sequences, and aligned in comparison to previously reported EBV strains (prototype B95-8, Med+, Med-, China 1, China 2, Alaskan, NC) using Clustal W method (Fig 3). Molecular Phylogenetic analysis by neighbor joining method and Kimura two-parameter algorithm were performed using CLC sequence viewer 7.7.1. software (Fig 4). Mediterranean strain was the most prevalent strain with a rate of 65.3% (32/ 49), 62.5% (20/32) of which were from Med- strain (without 30 bp deletion). Prototype B95.8 and NC strains were detected at lower rates, 12.2% each. The least represented was China 1 strain, which was detected in 3 individuals (6%). Interestingly, two of the samples (4%) had mixed infection, where Med- and B95.8 were detected. Table 6 summarizes the sub-genotypes in relation to the nationality of the donors.

**Table 6:** Distribution of EBV LMP-1 sub-genotypes by donors’ nationalities.

Nationality	Total No.	EBV LMP-1 strains				
		Med	NC	B95.8	China 1	Co-infection(Med and B95.8)
Qatar	14	5	2	4	1	2
Jordan	6	6				
Egypt	6	5	1			
India	6	5	1			
Syria	6	4	0	1	1	
Palestine	4	4	0			
Pakistan	3	2	1			
Iran KSA	2		1	1		
	1	1				
Germany	1				1	
Total	49	32	6	6	3	2

## Discussion

EBV is known to cause infectious mononucleosis, and contributes to the pathogenesis of several malignancies, including >95% of Burkitt’s lymphoma cases [46], 70–95% of Hodgkin’s disease [47], >90% of Non-Hodgkin’s disease [48], > 90% of lymphoepithelioma-like gastric carcinoma [49], 5–25% in gastric adenocarcinomas [48, 50] and >90% of lymphoproliferative disorders in immunocompromised individuals [48]. In a recent study [51], the global burden of EBV related deaths was estimated to be >140,000, accounting for 1.8% of all cancer fatalities. Accordingly, laboratories around the globe regularly screen for this virus considering its importance in cancer development especially in immunocompromised patients. In the Middle Eastern region, there are few studies that describe the epidemiology of the virus, and none have been conducted in Qatar. In this study, we report on the seroprevalence of EBV, and the circulating types and genotypes among healthy blood donors in Qatar.

We found high EBV seroprevalence among healthy blood donors (97.9%), which is similar to what have been reported worldwide [20, 52]. We found that 96% of blood donors less than 30 years old were EBV seropositive, and seropositivity rate increased to 100% in donors older than 40 years. Similar findings were reported in various studies including a new study from Thailand, where EBV seroprevalence increased with age reaching 100% for those above 40 years of age [26]. In another study conducted in the USA. in 2006, 94% of organ donors were EBV seropositive, with a lower rate being reported among young age groups (< / = 35 year) [53].

Due to the high number of seropositive samples (>90%), we found no significant difference in rates of seropositivity between donors from various nationalities. Rates among Egyptian, Indian and Pakistani nationalities in Qatar were similar to those reported in the donors’ countries of origin [5, 6, 54, 55].

Although the indirect immunofluorescence assay (IFA) is considered the gold standard for EBV immune-diagnosis, enzyme immunoassay (EIA) is routinely used in diagnostics because of its high throughput [56]. Such techniques in addition to others like ELISA are still preferable and commonly used despite their high degrees of variability [42]. Further, these techniques have been used to identify the stage of EBV infection by detecting antibodies to certain proteins, which could mislead diagnosis or produce false results.

EBNA-1 antibodies are usually used to identify past infections as they appear 3 to 6 months post infection [6, 57]. Nonetheless, 6.7% of the donors in our study that were categorized with past infection, based on the detection of VCA-IgG, were negative for EBNA-1 antibodies. This was not surprising considering that 5 to 10% of healthy people never develop EBNA-1 antibodies [58]. Moreover, in immunosuppressed patients, loss of anti EBNA-1 is frequently reported [59]. Cumulatively, [57]; our results supports and confirms other studies that the use VCA-IgG is superior to EBNA-1 IgG in detecting previous exposure to EBV, as VCA-IgG was found in all EBV seropositive individuals. [57].

On the other hand, IgG antibodies against EA are detected transiently in up to 3 months or more during infection mononucleosis [59]. When EA-IgG is detected after convalescence it indicates virus reactivation [60]. However, reactivation cannot be confirmed only by EA-IgG detection. Several studies have shown that around 20% of previously infected individuals retain EA-IgG for years [58]. Among our tested samples, 65 (9.7%) had detectable EA-IgG, and therefore, were classified under EBV reactivation category. Yet, even though EBV reactivation is not rare among healthy individuals and it can occur periodically, these findings need to be supported by other tests such as molecular testing [42], and by the clinical picture of the patient/donor to confirm a past infection with persistent EA-IgG.

VCA- IgM is produced transitionally and it is an indication of recent primary infection. Typically, VCA-IgM weans after convalescence (few weeks), and may persist for months [40], and generally, does not appear after that in lifetime [42]. Although VCA IgM appears early and helps in the diagnosis of acute EBV infection, there are some limitations concerning the interpretation of VCA IgM data as it was found that some acutely infected children and adults do not develop VCA-IgM [40]. Furthermore, EBV-IgM might cross-react with other antigenically related viruses, especially CMV [61, 62]. In the current study, 12 samples were positive for VCA-IgM using ELISA, however, they all tested positive with VCA-IgG avidity assay, indicating the presence of high avidity mature IgG antibodies, and eliminating the possibility of carrying an active infection. Avidity assay is therefore important to validate the serological profile and it has been found to be a reliable tool in EBV primary infection confirmation in patients with undetectable VCA-IgM and in the differential diagnosis as well [24, 63]. This differential serological profile, which has been reported in other studies as well, might be of clinical significance and shall be further studies.

At the molecular level, 52.6% of the samples had detectable EBV DNA as measured by the real-time PCR. High rates of EBV infection have been reported in healthy blood donors, reaching up to 72% in one of the studies [31]. In another study with larger number of samples, Nishiwaki et al. reported 39.5% viremia rate (377/953) in healthy individuals [64]. On the other hand, viremia rates could be as low as (5.1% as reported in some studies [65]. EBV detection by PCR is highly affected by the specimen used (whole blood versus PBMC versus serum) and the variation in sample types must always be considered when comparing different studies. Typically, healthy individuals do not carry EBV in their plasma, accordingly studies that screened for EBV in serum of healthy individuals reported very low or undetected rates [66–68].

Another advantage of EBV DNA detection is that it enables the differentiation between acute and silent reactivations [66, 69–71]. We therefore investigated the correlation of EBV viremia seroprevalence and the different antibodies in the blood. We also investigated the correlation of ELISA results with EBV viral load. PCR results confirmed the ELISA results of the seronegative group. On the other hand, only 60% of those classified with EBV reactivation were PCR positive. EBV reactivation is defined by the presence of past infection antibodies (VCA-IgG and EBNA1-IgG), combined with positivity of EA-IgG, which can also persist for lifetime in around 20% of individuals [42, 58]. Hence, serological testing only cannot confirm reactivation status or the exact reactivation time. Therefore, EBV DNA detection and viral load quantification is used to assist in the diagnosis of EBV reactivation, although discrepancies can be found between PCR and serology [69]. In our study, not all of the samples classified serologically with reactive EBV infection were positive by PCR. In addition, there was no significant difference when comparing EBV viral load between samples of different EBV infection stages. High viral load was expected in donors with reactivation status, but we recorded no significant difference when compared to those samples of past infection. This could be attributed to the small sample size of the reactivation group (n = 65). Moreover, we detected EBV in the isolated PBMC’s of blood donors rather than whole blood; hence, the viremia rates did not reflect the actual situation of the donors with EBV reactivation. Furthermore, our study is cross sectional and we only tested one sample point for each donor. Accordingly, there is a possibility that we could have missed the viremia phase in some donors.

Our study revealed that genotype 1 is predominant across different nationalities, with an average rate of 72.5%. This genotype is usually more prevalent in Europe, America, China, and South Asia [4, 15], compare to genotype 2 that is more prevalent in African and Papua New Guinean populations and that was detected in only in 23.5% of samples [6, 72]. Mixed infections were also detected in 2 out of 51 (3.9%) samples, which have been previously reported in several other studies [29] [9, 73], noting that infection with one genotype does not provide immunity against the other.

Further sub-genotypying analysis based on LMP-1 gene revealed the presence of four variants. Mediterranean strain was predominant (65.3%), with Med–being more prevalent than Med +; found in 65.1% of all Med isolates. Prototype strain B95.8 and North Carolina strain were found in 12.2%, and the least detected was China 1 strain (6%). Two samples (4%) showed the presence of two different strains Med and B95.8 indicating multiple EBV infections. Previous studies showed that the B95-8 prototype strain is typically prevalent in European isolates, whereas the China 1 strain appears to be the most prevalent in American and Asian isolates [13]. Mixed infection with more than one sub-genotype was previously reported where three strains China 1, B95.8 and Med were detected in a normal host [74]. We did not detect isolates that belonged to the SEA1, Alaskan, nor China 3 strains which are high-risk strains and are associated with EBV illnesses [12].

This is the first study that defined the status of EBV seroprevalence, genotypes, and sub-genotypes among healthy individual in Qatar and the Middle East. However, the study has some limitations. Firstly, we had few females (n = 14) participants compared to males (n = 659) and thus, rendering the comparison between two groups questionable. Moreover, the study was limited to samples collected from donors above 18 years of age, where most of participants were seropositive and a similar study with younger group is needed, especially when investigating EBV seroconversion, transmission, and vaccine administration.

In conclusion, our study corroborated previous studies that showed high seroprevalence of EBV, affecting more than 95% the population in Qatar. More importantly, we have shown a high viremia rate amongst participants (average of 52.6%), that significantly increased with age. EBV genotype 1, and sub-genotype Med were the predominant (72.5% and 65.3%, respectively) in the studied population. These data will increase awareness of EBV among researchers and healthcare workers in Qatar and will promote the adoption of safety practices in health care centers, especially in blood banks and organ transplant centers. Nonetheless, a wider analysis that includes younger participants, cancer patients, and patients with immunocompromised health conditions, will be necessary to estimate the risk of the virus in the population and to apply control and prevention measures when applicable.

## Supporting information

S1 Table List of patients’ information, preliminary serology results, and PCR results. (PDF)

S1 Fig Agarose gel electrophoresis results for the first round of PCR amplification for the EBNA2 gene using E2P1 and E2P2 primers that amplified almost the entire EBNA2 gene. (TIF)

S2 Fig Agarose gel electrophoresis results for the second round of PCR amplification for the EBNA2 gene using EBV type 1 specific primers (AP primers). (TIF)

S3 Fig Agarose gel electrophoresis results for the second round of PCR amplification for the EBNA2 gene using EBV type 2 specific primers (BP primers). (TIF)

S4 Fig Agarose gel electrophoresis results for the PCR amplification of the C- terminus of LMP-1 gene using primers that amplify a 587 bp fragment. (TIF)

S5 Fig Agarose gel electrophoresis results for *the Eco*RI restriction digestion of the pDrive cloning plasmids harboring the LMP-1 PCR fragment. The 587 bp cloned fragments (Lane 1–7) were separated after digestion of the pDrive plasmid (upper thick band) with *Eco*RI.
(TIF)
