# Supplementary material for: Prevalence and molecular profiling of Epstein Barr virus (EBV) among healthy blood donors from different nationalities in Qatar
Source: PLoS One. 2017 Dec 11;12(12):e0189033. doi: 10.1371/journal.pone.0189033 (PMC5724864; doi:10.1371/journal.pone.0189033)
Supplement: S1 Table — (PDF) [file pone.0189033.s001.pdf]

S1 Table. Donors' data sheet

Sample no.	Conc. ng/µl	EBV Copies/R	EBV Copies/ng	EBV Copies/ml	ELISA results	Gen.	Nation.	DOB	Age
**1**	19	0.0000	0.0000	0.00	Past Infection	M	EGY	1967	48
**2**	13	2.4810	0.0191	248.10	Reactive Infection	M	EGY	1973	42
**3**	34.8	1.4590	0.0042	145.90	Past Infection	M	PAL	1970	45
**4**	33.9	0.0000	0.0000	0.00	Past Infection	M	PAL	1964	51
**5**	69.6	3.0120	0.0043	301.20	Past Infection	M	IND	1980	35
**6**	90.6	0.0000	0.0000	0.00	Past Infection	M	JOR	1975	40
**7**	27.6	0.1830	0.0007	18.30	Past Infection	M	NEP	1986	29
**8**	12.2	0.0000	0.0000	0.00	Past Infection	M	PHI	1975	40
**9**	31.8	27.1390	0.0853	2713.90	Past Infection	M	IND	1984	31
**10**	11.1	11.3500	0.1023	1135.00	Past Infection	M	YEM	1990	25
**11**	4.2	3.1859	0.0759	318.59	Past Infection	M	PAK	1973	42
**23**	23	10.0230	0.0436	1002.30	Past Infection	M	SYR	1982	33
**24**	29.4	0.0000	0.0000	0.00	Past Infection	F	QAT	1994	21
**25**	15.8	1.0603	0.0067	106.03	Past Infection	M	EGY	1979	36
**26**	70.9	47.8940	0.0676	4789.40	Past Infection	M	SYR	1962	53
**28**	37.4	0.0000	0.0000	0.00	Past Infection	M	SYR	1987	28
**29**	16.1	1.9917	0.0124	199.17	Reactive Infection	M	IND	1965	50
**30**	29.2	10.5362	0.0361	1053.62	Past Infection	M	EGY	1987	28
**31**	14	0.0000	0.0000	0.00	Past Infection	M	PAK	1982	33
**32**	40.2	0.0000	0.0000	0.00	Past Infection	M	SYR	1988	27
**35**	36.6	0.0000	0.0000	0.00	Past Infection	M	YEM	1993	22
**70**	24	1.1894	0.0050	118.94	Past Infection	M	IND	1974	41
**71**	28.7	0.0000	0.0000	0.00	Past Infection	M	IND	1990	25
**72**	22	2.7977	0.0127	279.77	Past Infection	M	PAK	1956	59
**73**	22.7	3.6457	0.0161	364.57	Past Infection	M	EGY	1979	36

**74**	39.3	0.0000	0.0000	0.00	Past Infection	M	JOR	1981	34
**75**	23.8	2.9107	0.0122	291.07	Reactive Infection	M	QAT	1970	45
**76**	44.2	0.0000	0.0000	0.00	Past Infection	M	QAT	1983	32
**77**	48.1	1.3321	0.0028	133.21	Past Infection	M	SYR	1977	38
**78**	24.5	1.6549	0.0068	165.49	Reactive Infection	M	QAT	1980	35
**79**	10.4	0.0000	0.0000	0.00	Past Infection	M	SYR	1979	36
**80**	15.4	1.0298	0.0067	102.98	Reactive Infection	M	SYR	1982	33
**81**	54.1	0.0000	0.0000	0.00	Past Infection	M	EGY	1976	39
**82**	17.6	0.0000	0.0000	0.00	Past Infection	M	PAK	1971	44
**83**	41.3	4.2542	0.0103	425.42	Past Infection	M	JOR	1977	38
**84**	13.1	0.0000	0.0000	0.00	Past Infection	M	EGY	1971	44
**85**	31.8	1.1010	0.0035	110.10	Past Infection	M	IND	1975	40
**86**	26.4	2.7530	0.0104	275.30	Past Infection	M	JOR	1968	47
**87**	6.8	0.0000	0.0000	0.00	Past Infection	M	IND	1982	33
**88**	11.2	3.4040	0.0304	340.40	Past Infection	M	YEM	1989	26
**89**	6.1	0.0000	0.0000	0.00	Past Infection	M	QAT	1981	34
**90**	4.58	0.0000	0.0000	0.00	Past Infection	M	QAT	1987	28
**91**	5.4	3.7581	0.0696	375.81	Past Infection	M	SYR	1983	32
**92**	8.6	0.5000	0.0058	50.00	Reactive Infection	F	QAT	1993	22
**93**	11.1	1.5550	0.0140	155.50	Past Infection	M	SYR	1981	34
**94**	6.9	0.0000	0.0000	0.00	Past Infection	M	SYR	1984	31
**95**	7.5	4.9093	0.0655	490.93	Past Infection	M	QAT	1970	45
**96**	9.8	3.1268	0.0319	312.68	Past Infection	M	PAK	1973	42
**97**	10.7	0.0000	0.0000	0.00	Past Infection	M	JOR	1978	37
**98**	8.6	0.0000	0.0000	0.00	Past Infection	M	IND	1987	28
**99**	14.52	0.0000	0.0000	0.00	Past Infection	M	SYR	1985	30
**100**	32.4	10.8917	0.0336	1089.17	Past Infection	M	PAK	1974	41
**101**	47.4	0.0000	0.0000	0.00	Past Infection	M	YEM	1990	25
**102**	81.5	8.2259	0.0101	822.59	Active Infection	M	PAK	1975	40
**103**	35.5	0.0000	0.0000	0.00	Past Infection	M	QAT	1983	32

**104**	85.8	5.0896	0.0059	508.96	Past Infection	M	LEB	1992	23
**105**	65.5	22.4172	0.0342	2241.72	Past Infection	M	BAH	1986	29
**106**	109	130.5692	0.1198	13056.92	Past Infection	M	SYR	1965	50
**107**	47.8	0.0000	0.0000	0.00	Past Infection	M	QAT	1976	39
**108**	59.3	0.0000	0.0000	0.00	Reactive Infection	M	MOR	1976	39
**109**	94.7	4.1793	0.0044	417.93	Past Infection	M	QAT	1961	54
**110**	106	1.1660	0.0011	116.60	Reactive Infection	M	BAH	1967	48
**111**	28.9	0.0000	0.0000	0.00	Reactive Infection	M	IND	1969	46
**112**	25	205.8600	0.8234	20586.00	Reactive Infection	M	IND	1972	43
**113**	26	0.0000	0.0000	0.00	Past Infection	M	EGY	1988	27
**114**	20	0.0000	0.0000	0.00	Past Infection	M	LEB	1970	45
**115**	26	3.9007	0.0150	390.07	Reactive Infection	M	SYR	1993	22
**116**	19.9	0.0000	0.0000	0.00	Past Infection	M	IND	1983	32
**117**	23	8.6039	0.0374	860.39	Past Infection	M	EGY	1986	29
**119**	31.5	25.1101	0.0797	2511.01	Past Infection	M	JOR	1989	26
**120**	40	12.8915	0.0322	1289.15	Past Infection	M	JOR	1974	41
**121**	41.3	32.5428	0.0788	3254.28	Past Infection	M	PAK	1971	44
**122**	60	8.3663	0.0139	836.63	Past Infection	M	QAT	1965	50
**124**	50.9	0.0000	0.0000	0.00	Past Infection	M	SYR	1988	27
**126**	55	0.0000	0.0000	0.00	Past Infection	M	PAL	1965	50
**127**	68.1	5.0823	0.0075	508.23	Past Infection	M	SUD	1954	61
**128**	49.8	0.0000	0.0000	0.00	Past Infection	M	SYR	1981	34
**129**	40	0.0000	0.0000	0.00	Past Infection	M	QAT	1977	38
**130**	35	0.0000	0.0000	0.00	Past Infection	M	MOR	1984	31
**131**	133.4	17.5996	0.0132	1759.96	Past Infection	M	SUD	1986	29
**133**	58.5	0.0000	0.0000	0.00	Past Infection	M	IND	1989	26
**134**	66.8	0.0000	0.0000	0.00	Recent Active	M	EGY	1980	35
**135**	71.1	7.9998	0.0113	799.98	Past Infection	M	EGY	1977	38
**136**	19.9	0.0000	0.0000	0.00	Past Infection	M	SUD	1988	27

**137**	32.2	0.0000	0.0000	0.00	No Infection	M	SYR	1986	29
**138**	32.7	17.5773	0.0538	1757.73	Past Infection	M	SUD	1963	52
**139**	26.6	0.0000	0.0000	0.00	Past Infection	M	QAT	1982	33
**140**	24.1	16.2835	0.0676	1628.35	Past Infection	M	QAT	1967	48
**141**	54.6	0.0000	0.0000	0.00	Past Infection	M	PAK	1993	22
**142**	32.8	0.0000	0.0000	0.00	Past Infection	M	JOR	1970	45
**143**	40.2	3.5096	0.0087	350.96	Past Infection	M	JOR	1975	40
**144**	59	0.0000	0.0000	0.00	Past Infection	M	SYR	1986	29
**145**	39.3	9.0243	0.0230	902.43	Past Infection	M	QAT	1964	51
**146**	59.5	0.0000	0.0000	0.00	Past Infection	M	SYR	1976	39
**147**	49.6	0.0000	0.0000	0.00	Past Infection	M	IRN	1970	45
**148**	23.4	6.0302	0.0258	603.02	Past Infection	M	IND	1959	56
**149**	48.9	13.6118	0.0278	1361.18	Recent Active	M	SYR	1985	30
**150**	42.8	3.1285	0.0073	312.85	Past Infection	M	IND	1968	47
**151**	55	0.0000	0.0000	0.00	Past Infection	M	SYR	1989	26
**152**	88.5	0.0000	0.0000	0.00	Past Infection	M	JOR	1965	50
**153**	582.8	5.3296	0.0009	532.96	Past Infection	M	SYR	1983	32
**154**	35.8	0.0000	0.0000	0.00	No Infection	M	IND	1983	32
**155**	36	0.0000	0.0000	0.00	Past Infection	M	US	1958	57
**157**	60.4	0.0000	0.0000	0.00	Reactive Infection	M	PHI	1969	46
**158**	46.4	2.9478	0.0064	294.78	Past Infection	M	EGY	1977	38
**159**	37	79.8208	0.2157	7982.08	Reactive Infection	M	SYR	1990	25
**160**	37.9	3.1065	0.0082	310.65	Past Infection	M	YEM	1973	42
**161**	23	2.4538	0.0107	245.38	Past Infection	M	EGY	1973	42
**162**	53	7.5193	0.0142	751.93	Past Infection	M	LEB	1956	59
**163**	17.5	0.0000	0.0000	0.00	Past Infection	M	JOR	1966	49
**164**	37.8	4.6976	0.0124	469.76	Past Infection	M	EGY	1985	30
**165**	49.1	4.9651	0.0101	496.51	Past Infection	M	QAT	1988	27
**166**	57.2	13.6228	0.0238	1362.28	Past Infection	M	PHI	1974	41
**167**	47.3	60.5734	0.1281	6057.34	Past Infection	M	JOR	1969	46

**168**	31.2	0.0000	0.0000	0.00	Reactive Infection	M	JOR	1993	22
**169**	52.9	0.0000	0.0000	0.00	Past Infection	M	EGY	1984	31
**170**	70.3	5.5052	0.0078	550.52	Past Infection	M	PHI	1988	27
**171**	46.5	0.0000	0.0000	0.00	Past Infection	M	SYR	1978	37
**172**	94	0.0000	0.0000	0.00	Past Infection	M	SYR	1981	34
**173**	63.7	0.0000	0.0000	0.00	Past Infection	M	QAT	1960	55
**174**	36.4	0.0000	0.0000	0.00	Past Infection	M	QAT	1977	38
**175**	39	0.0000	0.0000	0.00	Past Infection	M	SYR	1984	31
**176**	47.2	3.1035	0.0066	310.35	Reactive Infection	M	SYR	1972	43
**177**	160.2	2.6013	0.0016	260.13	Past Infection	M	EGY	1982	33
**178**	37.9	0.0000	0.0000	0.00	Past Infection	M	QAT	1983	32
**179**	41.7	0.0000	0.0000	0.00	Past Infection	M	SUD	1991	24
**180**	40.4	11.4726	0.0284	1147.26	Past Infection	M	EGY	1989	26
**181**	216.5	2.7404	0.0013	274.04	Past Infection	M	EGY	1979	36
**182**	59.7	3.0253	0.0051	302.53	Active Infection	M	SUD	1988	27
**183**	56.2	4.1341	0.0074	413.41	Past Infection	M	LEB	1986	29
**184**	41.1	3.4765	0.0085	347.65	Past Infection	M	LEB	1971	44
**185**	84.7	0.0000	0.0000	0.00	Past Infection	M	PHI	1981	34
**186**	63.5	0.0000	0.0000	0.00	Past Infection	F	LEB	1984	31
**187**	79.9	14.3280	0.0179	1432.80	Past Infection	M	GER	1966	49
**188**	64.3	2.0710	0.0032	207.10	Reactive Infection	F	US	1970	45
**189**	42.6	2.4349	0.0057	243.49	Reactive Infection	F	AUS	1972	43
**190**	50.1	0.0000	0.0000	0.00	No Infection	M	SYR	1976	39
**191**	32.3	13.6329	0.0422	1363.29	Past Infection	M	JOR	1965	50
**192**	60.5	0.0000	0.0000	0.00	Past Infection	M	QAT	1978	37
**193**	75.3	0.0000	0.0000	0.00	Past Infection	M	EGY	1982	33
**195**	66.8	4.5746	0.0068	457.46	Past Infection	M	IND	1968	47
**196**	61.4	0.0000	0.0000	0.00	Past Infection	M	PAL	1968	47
**197**	43.5	11.8220	0.0272	1182.20	Past Infection	M	EGY	1983	32
**199**	51.6	7.7879	0.0151	778.79	Past Infection	M	EGY	1975	40

**200**	64	0.0000	0.0000	0.00	Past Infection	M	QAT	1996	19
**201**	77.6	0.0000	0.0000	0.00	Past Infection	M	QAT	1983	32
**202**	59.2	0.0000	0.0000	0.00	Past Infection	M	SYR	1988	27
**203**	63.9	5.6775	0.0089	567.75	Past Infection	M	PAL	1973	42
**204**	46.9	0.0000	0.0000	0.00	Past Infection	M	SYR	1964	51
**205**	67	0.0000	0.0000	0.00	Past Infection	M	IND	1976	39
**206**	28.8	0.0000	0.0000	0.00	Past Infection	M	EGY	1980	35
**207**	35.8	2.4498	0.0068	244.98	Past Infection	M	BAN	1988	27
**208**	16.4	2.9383	0.0179	293.83	Past Infection	M	CAN	1970	45
**209**	24.9	0.0000	0.0000	0.00	Past Infection	M	IND	1986	29
**210**	35.4	0.6000	0.0017	60.00	Reactive Infection	M	NEP	1985	30
**211**	46.5	2.9482	0.0063	294.82	Past Infection	M	US	1975	40
**212**	33.6	13.0748	0.0389	1307.48	Early Infection	M	Cyprus	1996	19
**213**	22	0.0000	0.0000	0.00	Past Infection	M	ITA	1978	37
**214**	18.1	0.0000	0.0000	0.00	Past Infection	M	EGY	1988	27
**215**	1809.1	7.1411	0.0004	714.11	Past Infection	M	QAT	1982	33
**216**	39.2	18.5852	0.0474	1858.52	Past Infection	M	QAT	1969	46
**217**	47.2	0.0000	0.0000	0.00	Past Infection	M	EGY	1983	32
**218**	33.1	9.0705	0.0274	907.05	Past Infection	M	SYR	1982	33
**219**	34.6	0.0000	0.0000	0.00	Past Infection	M	EGY	1982	33
**220**	68.7	0.0000	0.0000	0.00	Past Infection	M	IND	1974	41
**221**	75.2	11.2010	0.0149	1120.10	Past Infection	M	EGY	1961	54
**222**	62.9	7.9416	0.0126	794.16	Reactive Infection	M	QAT	1973	42
**223**	61.6	0.0000	0.0000	0.00	Past Infection	M	QAT	1978	37
**224**	33.3	4.4009	0.0132	440.09	Past Infection	M	SYR	1982	33
**225**	42	38.3475	0.0913	3834.75	Past Infection	M	EGY	1965	50
**226**	17.8	2.9693	0.0167	296.93	Past Infection	M	QAT	1972	43
**227**	34.6	20.8490	0.0603	2084.90	Past Infection	M	QAT	1973	42
**228**	90.5	2.7660	0.0031	276.60	Past Infection	M	PAL	1983	32
**229**	38.4	4.9599	0.0129	495.99	Past Infection	M	SYR	1959	56

**230**	33.2	0.0000	0.0000	0.00	Past Infection	M	EGY	1972	43
**231**	28.4	0.0000	0.0000	0.00	Past Infection	M	QAT	1987	28
**232**	13.5	2.6070	0.0193	260.70	Past Infection	M	EGY	1986	29
**233**	23.1	0.0000	0.0000	0.00	Past Infection	M	PAK	1972	43
**234**	50	0.0000	0.0000	0.00	Past Infection	M	SYR	1986	29
**235**	18.7	2.9011	0.0155	290.11	Past Infection	M	YEM	1982	33
**236**	17.2	0.0000	0.0000	0.00	Past Infection	M	SYR	1974	41
**237**	17	0.0000	0.0000	0.00	Past Infection	M	EGY	1982	33
**238**	48.1	3.1839	0.0066	318.39	Past Infection	M	SYR	1962	53
**239**	21.2	12.0699	0.0569	1206.99	Past Infection	M	UK	1968	47
**240**	9	2.4849	0.0276	248.49	Past Infection	M	EGY	1986	29
**241**	30.2	0.0000	0.0000	0.00	Past Infection	M	TUN	1972	43
**242**	15.5	0.0000	0.0000	0.00	Past Infection	M	EGY	1985	30
**243**	14.6	0.0000	0.0000	0.00	Past Infection	M	JOR	1962	53
**244**	19.1	0.0000	0.0000	0.00	Past Infection	M	EGY	1991	24
**245**	17.6	0.0000	0.0000	0.00	Past Infection	M	QAT	1972	43
**246**	23.2	0.0000	0.0000	0.00	Reactive Infection	M	SYR	1993	22
**247**	19.9	0.0000	0.0000	0.00	Past Infection	M	EGY	1970	45
**248**	24.9	20.8376	0.0837	2083.76	Past Infection	M	SYR	1980	35
**249**	56.5	0.0000	0.0000	0.00	Past Infection	M	SYR	1980	35
**250**	54.9	0.0000	0.0000	0.00	Reactive Infection	M	SYR	1989	26
**251**	56.5	0.0000	0.0000	0.00	Past Infection	M	QAT	1974	41
**252**	39	0.0000	0.0000	0.00	Past Infection	M	PAL	1995	20
**253**	25.6	0.0000	0.0000	0.00	No Infection	M	OMAN	1987	28
**254**	41.2	0.0000	0.0000	0.00	Past Infection	M	SYR	1992	23
**255**	20.9	43.4738	0.2080	4347.38	Past Infection	M	QAT	1978	37
**256**	45.1	0.0000	0.0000	0.00	Past Infection	M	QAT	1959	56
**257**	88	13.9614	0.0159	1396.14	Past Infection	M	IRQ	1968	47
**258**	41.8	2.9198	0.0070	291.98	Past Infection	M	QAT	1987	28
**259**	33.5	2.2147	0.0066	221.47	Past Infection	M	SYR	1980	35

**260**	42.9	0.0000	0.0000	0.00	Past Infection	M	PAL	1975	40
**261**	48.7	12.2499	0.0252	1224.99	Past Infection	M	JOR	1979	36
**262**	16.8	5.3863	0.0321	538.63	Past Infection	M	IND	1986	29
**263**	25.1	0.0000	0.0000	0.00	Past Infection	M	EGY	1981	34
**264**	42.2	0.0000	0.0000	0.00	Past Infection	M	ALG	1989	26
**265**	35.4	0.0000	0.0000	0.00	Past Infection	F	EGY	1981	34
**266**	20.8	0.0000	0.0000	0.00	Past Infection	M	IND	1985	30
**267**	25.1	3.1000	0.0124	310.00	Reactive Infection	M	IND	1975	40
**268**	24.5	0.0000	0.0000	0.00	Past Infection	M	QAT	1965	50
**269**	27.6	0.0000	0.0000	0.00	Reactive Infection	M	QAT	1970	45
**270**	22.3	0.0000	0.0000	0.00	Past Infection	M	SYR	1977	38
**271**	41.2	0.0000	0.0000	0.00	Past Infection	M	SYR	1991	24
**272**	38.4	12.5921	0.0328	1259.21	Past Infection	M	IND	1975	40
**273**	37.5	0.0000	0.0000	0.00	Past Infection	M	EGY	1984	31
**274**	36.4	0.0000	0.0000	0.00	Past Infection	M	IRN	1976	39
**275**	32.5	11.2784	0.0347	1127.84	Past Infection	M	JOR	1960	55
**276**	23.6	0.0000	0.0000	0.00	Past Infection	M	QAT	1966	49
**277**	20.3	0.0000	0.0000	0.00	Past Infection	M	JOR	1967	48
**278**	22.3	2.8794	0.0129	287.94	Past Infection	M	PAK	1967	48
**279**	37.5	0.0000	0.0000	0.00	Past Infection	M	SUD	1974	41
**280**	17.5	0.0000	0.0000	0.00	Past Infection	M	QAT	1981	34
**281**	23	0.0000	0.0000	0.00	Past Infection	M	EGY	1990	25
**282**	30.1	0.0000	0.0000	0.00	Past Infection	M	EGY	1987	28
**283**	28.5	0.0000	0.0000	0.00	No Infection	M	PAL	1984	31
**284**	15.5	0.0000	0.0000	0.00	Past Infection	M	QAT	1974	41
**285**	31	2.4158	0.0078	241.58	Past Infection	M	SYR	1982	33
**286**	21.2	0.0000	0.0000	0.00	Past Infection	M	LEB	1980	35
**287**	17.7	0.0000	0.0000	0.00	Past Infection	M	EGY	1984	31
**289**	10.4	0.0000	0.0000	0.00	Past Infection	M	EGY	1986	29
**290**	51.4	1.0540	0.0021	105.40	Reactive Infection	M	EGY	1981	34

**291**	22.3	2.7423	0.0123	274.23	Past Infection	M	IND	1970	45
**292**	11.1	2.5955	0.0234	259.55	Past Infection	M	JOR	1974	41
**293**	29.4	0.0000	0.0000	0.00	Recent Active	M	SYR	1979	36
**294**	22	0.4770	0.0022	47.70	Active Infection	M	QAT	1986	29
**295**	16.8	0.9443	0.0056	94.43	Past Infection	M	QAT	1976	39
**296**	24.7	0.0000	0.0000	0.00	Past Infection	M	EGY	1980	35
**297**	18.8	1.3085	0.0070	130.85	Past Infection	M	EGY	1975	40
**298**	11.8	0.0000	0.0000	0.00	Past Infection	M	SYR	1972	43
**299**	12.9	0.0000	0.0000	0.00	Reactive Infection	F	EGY	1994	21
**301**	6.2	0.0000	0.0000	0.00	Past Infection	M	JOR	1987	28
**303**	12.1	0.0000	0.0000	0.00	Past Infection	M	QAT	1986	29
**304**	16.2	0.0000	0.0000	0.00	Past Infection	M	PAL	1973	42
**305**	11.7	5.5443	0.0474	554.43	Past Infection	M	SYR	1983	32
**306**	17.9	0.0000	0.0000	0.00	Past Infection	M	IRN	1994	21
**307**	20.1	0.0000	0.0000	0.00	Past Infection	M	EGY	1967	48
**308**	22.4	0.0000	0.0000	0.00	Past Infection	M	PAL	1985	30
**309**	7.4	0.0000	0.0000	0.00	Past Infection	M	YEM	1982	33
**311**	37.7	0.0000	0.0000	0.00	Past Infection	M	BAN	1974	41
**312**	28.7	10.0523	0.0350	1005.23	Past Infection	M	SYR	1982	33
**313**	26.2	4.9064	0.0187	490.64	Past Infection	M	SUD	1977	38
**314**	10.2	0.0000	0.0000	0.00	Past Infection	M	QAT	1972	43
**315**	22	0.0000	0.0000	0.00	Past Infection	M	SYR	1980	35
**316**	44	0.0000	0.0000	0.00	Past Infection	M	NEP	1988	27
**317**	67.9	0.0000	0.0000	0.00	Past Infection	M	BAN	1988	27
**318**	46.9	0.0000	0.0000	0.00	Past Infection	M	EGY	1982	33
**319**	46	6.7969	0.0148	679.69	Past Infection	M	QAT	1960	55
**320**	56.5	0.0000	0.0000	0.00	Past Infection	M	QAT	1977	38
**321**	47.6	0.8516	0.0018	85.16	Past Infection	M	PAK	1991	24
**322**	34.7	29.3651	0.0846	2936.51	Past Infection	M	LEB	1987	28
**323**	53	0.0000	0.0000	0.00	Past Infection	M	IRQ	1967	48

**324**	59.1	1.0639	0.0018	106.39	Past Infection	M	QAT	1974	41
**325**	19.6	0.0000	0.0000	0.00	Past Infection	M	LEB	1982	33
**326**	44.6	4.5491	0.0102	454.91	Past Infection	M	QAT	1989	26
**327**	24	0.0000	0.0000	0.00	Reactive Infection	M	SUD	1988	27
**328**	43.9	2.8327	0.0065	283.27	Past Infection	M	SUD	1954	61
**329**	39.4	7.9335	0.0201	793.35	Past Infection	M	IRQ	1988	27
**330**	50.7	0.0000	0.0000	0.00	Past Infection	M	PAL	1957	58
**331**	53.6	0.0000	0.0000	0.00	Past Infection	M	SYR	1981	34
**332**	61.1	0.0000	0.0000	0.00	Past Infection	M	OMAN	1976	39
**333**	49.8	1.6656	0.0033	166.56	Past Infection	M	SYR	1970	45
**334**	47.1	1.7109	0.0036	171.09	Past Infection	M	SAUDI	1994	21
**335**	43.7	4.3837	0.0100	438.37	Past Infection	M	EGY	1989	26
**336**	23.3	1.8813	0.0081	188.13	Past Infection	M	QAT	1985	30
**337**	33.8	0.0000	0.0000	0.00	Reactive Infection	M	SYR	1977	38
**338**	29.5	1.6671	0.0057	166.71	Past Infection	M	EGY	1984	31
**339**	54.4	0.0000	0.0000	0.00	No Infection	M	AUS	1977	38
**341**	80	0.0000	0.0000	0.00	Past Infection	M	SYR	1981	34
**342**	72.4	9.3654	0.0129	936.54	Past Infection	M	EGY	1969	46
**343**	48.2	2.2904	0.0048	229.04	Past Infection	M	LEB	1982	33
**344**	57	1.7015	0.0030	170.15	Past Infection	M	SYR	1975	40
**345**	65.2	2.0154	0.0031	201.54	Past Infection	M	EGY	1980	35
**346**	220	1.8322	0.0008	183.22	Past Infection	M	ERITERIA	1976	39
**347**	33.4	7.5767	0.0227	757.67	Past Infection	M	QAT	1978	37
**348**	26.6	0.0000	0.0000	0.00	Past Infection	M	QAT	1963	52
**349**	59.5	0.0000	0.0000	0.00	Past Infection	M	EGY	1970	45
**350**	48.2	0.0000	0.0000	0.00	No Infection	M	JOR	1989	26
**351**	38.9	0.0000	0.0000	0.00	Reactive Infection	M	YEM	1981	34
**352**	34.7	0.0000	0.0000	0.00	Past Infection	M	QAT	1977	38
**353**	11.9	0.0000	0.0000	0.00	Reactive Infection	M	PAL	1973	42
**354**	22.7	46.084	0.2030	4608.37	Past Infection	M	QAT	1969	46

**355**	34.9	37.8201	0.1084	3782.01	Past Infection	M	PAL	1980	35
**356**	24.2	1.3406	0.0055	134.06	Reactive Infection	M	IND	1989	26
**357**	0	0.0000	0.0000	0.00	Past Infection	M	QAT	1968	47
**358**	11.6	0.0000	0.0000	0.00	Recent Active	M	QAT	1955	60
**359**	15.5	0.0000	0.0000	0.00	Past Infection	M	QAT	1982	33
**360**	20.3	1.4196	0.0070	141.96	Past Infection	M	MOR	1985	30
**361**	25.6	0.0000	0.0000	0.00	Past Infection	M	SYR	1957	58
**362**	26.6	0.0000	0.0000	0.00	Past Infection	M	EGY	1987	28
**364**	33.6	0.0000	0.0000	0.00	Past Infection	M	QAT	1987	28
**365**	32.3	0.0000	0.0000	0.00	Past Infection	M	QAT	1963	52
**366**	40.5	1.2096	0.0030	120.96	Past Infection	M	SYR	1986	29
**367**	38.8	4.6254	0.0119	462.54	Past Infection	M	SYR	1984	31
**368**	38.2	0.0000	0.0000	0.00	Past Infection	M	IND	1970	45
**369**	37.2	1.1478	0.0031	114.78	Past Infection	M	SYR	1988	27
**370**	36.1	0.0000	0.0000	0.00	Past Infection	M	PAK	1969	46
**371**	39.9	16.3739	0.0410	1637.39	Past Infection	M	QAT	1961	54
**372**	14.5	4.5875	0.0316	458.75	Past Infection	M	QAT	1960	55
**373**	22.6	1.6085	0.0071	160.85	Past Infection	M	FRA	1980	35
**374**	27.7	1.1163	0.0040	111.63	Past Infection	M	QAT	1969	46
**376**	14.4	2.6296	0.0183	262.96	Past Infection	M	PAL	1964	51
**377**	43	187.2940	0.4356	18729.40	Active Infection	M	SYR	1965	50
**378**	50.8	26.0108	0.0512	2601.08	Past Infection	F	SYR	1974	41
**379**	26.1	2.2409	0.0086	224.09	Past Infection	M	YEM	1977	38
**380**	21.3	0.0000	0.0000	0.00	Reactive Infection	M	SYR	1980	35
**381**	32.5	2.7885	0.0086	278.85	Past Infection	M	EGY	1974	41
**382**	44.3	0.0000	0.0000	0.00	Past Infection	M	IND	1983	32
**383**	30.8	0.0000	0.0000	0.00	Past Infection	M	LEB	1987	28
**384**	23.9	0.0000	0.0000	0.00	Past Infection	M	EGY	1971	44
**385**	36.7	0.0000	0.0000	0.00	Reactive Infection	M	CAN	1975	40
**386**	40.2	0.0000	0.0000	0.00	Past Infection	M	QAT	1969	46

**387**	23.8	0.0000	0.0000	0.00	Reactive Infection	M	EGY	1975	40
**388**	62.8	7.2658	0.0116	726.58	Past Infection	M	EGY	1977	38
**389**	20.6	0.0000	0.0000	0.00	Past Infection	M	QAT	1989	26
**390**	26	0.6854	0.0026	68.54	Past Infection	M	IND	1957	58
**391**	38.2	10.2255	0.0268	1022.55	Past Infection	M	EGY	1981	34
**392**	14.8	2.3364	0.0158	233.64	Active Infection	M	EGY	1972	43
**393**	50.1	3.8531	0.0077	385.31	Past Infection	M	JOR	1988	27
**394**	53	2.0946	0.0040	209.46	Past Infection	M	PAK	1977	38
**395**	40.4	3.6074	0.0089	360.74	Past Infection	M	PAL	1969	46
**396**	40.9	2.3614	0.0058	236.14	Past Infection	M	PAL	1955	60
**397**	58.6	14.1400	0.0241	1414.00	Past Infection	M	EGY	1968	47
**398**	37.8	6.5764	0.0174	657.64	Past Infection	M	LEB	1985	30
**399**	52.1	3.6294	0.0070	362.94	Past Infection	M	PAK	1982	33
**400**	28.6	1.4988	0.0052	149.88	Past Infection	M	GREECE	1976	39
**401**	34.4	0.0000	0.0000	0.00	No Infection	M	JOR	1989	26
**402**	64.6	3.5460	0.0055	354.60	Past Infection	M	IND	1982	33
**403**	82.8	21.0599	0.0254	2105.99	Past Infection	M	PAK	1983	32
**404**	95.9	8.0973	0.0084	809.73	Past Infection	M	US	1972	43
**405**	63.4	0.0000	0.0000	0.00	Past Infection	M	IND	1985	30
**406**	28.4	1.8568	0.0065	185.68	Past Infection	M	EGY	1982	33
**407**	40.6	0.0000	0.0000	0.00	Past Infection	M	EGY	1968	47
**408**	37.5	3.8522	0.0103	385.22	Past Infection	M	SYR	1979	36
**409**	63.8	86.4255	0.1355	8642.55	Past Infection	M	IRN	1989	26
**410**	29.4	0.0000	0.0000	0.00	Past Infection	M	SYR	1980	35
**411**	32.6	0.0000	0.0000	0.00	Past Infection	M	SYR	1970	45
**412**	26.1	0.0000	0.0000	0.00	Past Infection	M	IRN	1995	20
**413**	25.8	1.6746	0.0065	167.46	Past Infection	M	SYR	1983	32
**414**	65.1	0.0000	0.0000	0.00	Past Infection	M	SYR	1977	38
**415**	43.3	0.0000	0.0000	0.00	Past Infection	M	QAT	1991	24
**416**	28.1	0.0000	0.0000	0.00	Past Infection	M	IRN	1970	45

**417**	30.2	0.0000	0.0000	0.00	Past Infection	M	SYR	1983	32
**418**	53	0.0000	0.0000	0.00	Past Infection	M	IND	1989	26
**419**	38.2	2.8397	0.0074	283.97	Past Infection	M	SYR	1980	35
**420**	50.8	0.0000	0.0000	0.00	Past Infection	M	SYR	1974	41
**421**	55.3	6.0881	0.0110	608.81	Past Infection	M	JOR	1979	36
**422**	37.8	0.0000	0.0000	0.00	Past Infection	M	EGY	1975	40
**423**	63.6	0.0000	0.0000	0.00	Past Infection	M	UK	1984	31
**424**	35	0.0000	0.0000	0.00	Reactive Infection	M	PHI	1979	36
**426**	71.1	29.9942	0.0422	2999.42	Past Infection	M	SYR	1989	26
**427**	80.7	0.0000	0.0000	0.00	Past Infection	M	SRL	1981	34
**428**	80.6	3.7764	0.0047	377.64	Past Infection	M	EGY	1976	39
**429**	68.6	1.6737	0.0024	167.37	Past Infection	M	SYR	1967	48
**430**	83.3	2.9514	0.0035	295.14	Past Infection	M	QAT	1988	27
**431**	79.3	0.0000	0.0000	0.00	Past Infection	M	QAT	1974	41
**432**	98.9	66.6484	0.0674	6664.84	Past Infection	M	IRN	1969	46
**433**	49.6	1.7398	0.0035	173.98	Past Infection	M	QAT	1980	35
**434**	120.2	3.2846	0.0027	328.46	Reactive Infection	M	QAT	1959	56
**435**	86.9	0.0000	0.0000	0.00	Reactive Infection	M	YEM	1970	45
**436**	84	0.0000	0.0000	0.00	Reactive Infection	M	SYR	1986	29
**437**	50.7	9.9341	0.0196	993.41	Past Infection	M	EGY	1982	33
**438**	65.2	0.0000	0.0000	0.00	Past Infection	M	QAT	1977	38
**439**	55.1	0.0000	0.0000	0.00	Past Infection	M	SRL	1969	46
**440**	44.1	1.8498	0.0042	184.98	Past Infection	M	EGY	1979	36
**441**	101.6	1.4673	0.0014	146.73	Past Infection	M	SYR	1968	47
**442**	60.5	0.0000	0.0000	0.00	Past Infection	M	LEB	1974	41
**443**	47.8	2.8106	0.0059	281.06	Past Infection	M	CROATIA	1981	34
**444**	80	0.0000	0.0000	0.00	Past Infection	M	SYR	1982	33
**445**	87.3	2.8470	0.0033	284.70	Past Infection	M	SYR	1975	40
**446**	77.1	0.0000	0.0000	0.00	Reactive Infection	F	EGY	1991	24
**447**	49.4	0.0000	0.0000	0.00	Past Infection	F	QAT	1996	19

**448**	50.1	2.0350	0.0041	203.50	Active Infection	M	IND	1977	38
**449**	81.7	0.0000	0.0000	0.00	Past Infection	M	BAN	1994	21
**450**	63.3	0.0000	0.0000	0.00	Past Infection	M	BAN	1968	47
**451**	63.4	1.4692	0.0023	146.92	Past Infection	M	BAN	1962	53
**452**	78.1	0.0000	0.0000	0.00	Past Infection	M	BAN	1986	29
**453**	91.2	2.7352	0.0030	273.52	Past Infection	M	BAN	1973	42
**454**	54.6	0.0000	0.0000	0.00	Past Infection	M	SRL	1980	35
**455**	49.4	29.8431	0.0604	2984.31	Past Infection	M	SAUDI	1991	24
**456**	53.4	0.0000	0.0000	0.00	Past Infection	M	IND	1984	31
**457**	73.7	0.0000	0.0000	0.00	Reactive Infection	M	UK	1963	52
**458**	61.4	0.0000	0.0000	0.00	Past Infection	M	IND	1989	26
**459**	55.8	1.5673	0.0028	156.73	Past Infection	M	SYR	1978	37
**460**	66.6	0.0000	0.0000	0.00	Past Infection	M	IND	1989	26
**461**	52.4	1.8266	0.0035	182.66	Past Infection	M	IND	1985	30
**462**	66.6	1.7361	0.0026	173.61	Past Infection	M	QAT	1970	45
**463**	67.9	2.1362	0.0031	213.62	Past Infection	M	QAT	1975	40
**464**	70.4	0.0000	0.0000	0.00	Past Infection	M	JOR	1978	37
**465**	83.8	0.0000	0.0000	0.00	Past Infection	M	PHI	1961	54
**466**	68.8	14.5561	0.0212	1455.61	Past Infection	M	PAL	1968	47
**467**	78.8	3.0620	0.0039	306.20	Past Infection	M	PAL	1993	22
**468**	58.1	0.0000	0.0000	0.00	No Infection	M	QAT	1991	24
**469**	110.1	7.8664	0.0071	786.64	Past Infection	M	BAN	1993	22
**470**	90.7	0.0000	0.0000	0.00	Past Infection	F	PHI	1993	22
**471**	67.3	3.3560	0.0050	335.60	Past Infection	M	PAL	1973	42
**472**	94.7	3.2556	0.0034	325.56	Past Infection	M	QAT	1969	46
**473**	110.3	3.9432	0.0036	394.32	Past Infection	M	EGY	1973	42
**474**	182.2	11.2361	0.0062	1123.61	Past Infection	M	EGY	1975	40
**475**	149.9	0.0000	0.0000	0.00	Past Infection	M	IND	1985	30
**476**	132.8	23.0670	0.0174	2306.70	Past Infection	M	EGY	1990	25
**477**	84.4	5.7746	0.0068	577.46	Past Infection	M	SAUDI	1950	65

**478**	126.4	2.2954	0.0018	229.54	Past Infection	M	QAT	1969	46
**479**	115.1	0.0000	0.0000	0.00	Past Infection	M	IND	1973	42
**480**	112.1	3.2142	0.0029	321.42	Past Infection	M	IND	1992	23
**481**	136.4	0.0000	0.0000	0.00	Past Infection	M	IND	1967	48
**482**	102.6	0.0000	0.0000	0.00	Past Infection	M	NEP	1982	33
**483**	43.8	0.0000	0.0000	0.00	Past Infection	M	PAL	1973	42
**484**	119.3	7.8623	0.0066	786.23	Past Infection	M	EGY	1985	30
**485**	143.6	3.4666	0.0024	346.66	Past Infection	M	IND	1986	29
**486**	95.7	0.0000	0.0000	0.00	Past Infection	M	PAL	1972	43
**487**	104.1	0.0000	0.0000	0.00	Past Infection	M	NEP	1979	36
**488**	94	0.0000	0.0000	0.00	Past Infection	M	SUD	1988	27
**489**	138.8	8.7033	0.0063	870.33	Past Infection	M	IND	1983	32
**490**	90.6	2.4732	0.0027	247.32	Past Infection	M	SYR	1975	40
**491**	102.3	33.6520	0.0329	3365.20	Past Infection	M	PAK	1975	40
**492**	149.4	2.1493	0.0014	214.93	Past Infection	M	BAN	1986	29
**493**	86.9	0.0000	0.0000	0.00	Past Infection	M	QAT	1981	34
**494**	129.7	1.4419	0.0011	144.19	Past Infection	M	SRL	1985	30
**495**	31.8	0.0000	0.0000	0.00	Past Infection	M	QAT	1993	22
**496**	105.9	0.0000	0.0000	0.00	Past Infection	M	IND	1971	44
**497**	109.8	8.9340	0.0081	893.40	Past Infection	M	SYR	1990	25
**498**	123.2	0.0000	0.0000	0.00	Past Infection	M	QAT	1982	33
**499**	78.6	0.0000	0.0000	0.00	Past Infection	M	QAT	1973	42
**500**	54.8	29.2937	0.0535	2929.37	Past Infection	M	QAT	1954	61
**501**	76.4	0.0000	0.0000	0.00	Past Infection	M	QAT	1963	52
**502**	52.4	0.0000	0.0000	0.00	Past Infection	M	QAT	1978	37
**503**	70.9	0.0000	0.0000	0.00	Past Infection	M	SRL	1972	43
**504**	91.9	11.1446	0.0121	1114.46	Past Infection	M	EGY	1984	31
**505**	47.4	1.2661	0.0027	126.61	Past Infection	M	EGY	1984	31
**506**	76.9	0.0000	0.0000	0.00	Past Infection	M	EGY	1981	34
**507**	84	0.2391	0.0003	23.91	Past Infection	M	QAT	1977	38

**508**	112.3	8.7330	0.0078	873.30	Past Infection	M	QAT	1984	31
**509**	99.3	1.1780	0.0012	117.80	Reactive Infection	M	SUD	1975	40
**510**	48.4	0.0000	0.0000	0.00	Past Infection	M	QAT	1989	26
**511**	83.3	2.8285	0.0034	282.85	Past Infection	M	IND	1983	32
**512**	50.3	0.0000	0.0000	0.00	Past Infection	M	SYR	1983	32
**513**	40	0.0000	0.0000	0.00	Past Infection	M	IND	1982	33
**514**	55.4	0.0000	0.0000	0.00	Past Infection	M	IND	1988	27
**515**	289.2	5.3303	0.0018	533.03	Past Infection	M	IND	1987	28
**516**	50.2	0.0000	0.0000	0.00	Reactive Infection	M	IND	1984	31
**517**	39.8	0.0000	0.0000	0.00	Past Infection	M	PAL	1984	31
**518**	55.8	1.4214	0.0025	142.14	Past Infection	M	LEB	1988	27
**519**	50.4	0.0000	0.0000	0.00	Past Infection	M	PAL	1979	36
**520**	48.4	0.0000	0.0000	0.00	Past Infection	M	IND	1966	49
**521**	63.1	1.3346	0.0021	133.46	Past Infection	M	QAT	1989	26
**522**	48.7	0.0000	0.0000	0.00	Past Infection	M	ECUADUR	1987	28
**523**	66.2	2.1436	0.0032	214.36	Past Infection	M	SYR	1981	34
**524**	56.4	0.0000	0.0000	0.00	Reactive Infection	M	EGY	1973	42
**525**	34.1	0.0000	0.0000	0.00	Past Infection	M	EGY	1977	38
**526**	39.1	13.2300	0.0338	1323.00	Past Infection	M	IND	1987	28
**527**	71.1	3.3120	0.0047	331.20	Reactive Infection	M	QAT	1972	43
**528**	45.2	11.6030	0.0257	1160.30	Past Infection	M	EGY	1977	38
**529**	62.9	5.8700	0.0093	587.00	Past Infection	M	JOR	1963	52
**530**	65.2	8.9600	0.0137	896.00	Past Infection	M	SYR	1980	35
**531**	75.6	0.0000	0.0000	0.00	Past Infection	M	IND	1988	27
**532**	79.5	35.2700	0.0444	3527.00	Reactive Infection	M	NEP	1977	38
**533**	79.4	0.0000	0.0000	0.00	Past Infection	M	PHI	1977	38
**534**	76.4	16.4230	0.0215	1642.30	Past Infection	M	IND	1978	37
**535**	52.4	1.3160	0.0025	131.60	Reactive Infection	M	ETH	1985	30
**536**	70.9	0.0000	0.0000	0.00	Past Infection	M	IRN	1966	49
**537**	91.9	229.7500	0.2500	22975.00	Past Infection	M	QAT	1983	32

**538**	47.4	1.0430	0.0022	104.30	Early Infection	M	PAK	1976	39
**539**	76.9	0.0000	0.0000	0.00	Past Infection	M	PAL	1979	36
**541**	84	0.0000	0.0000	0.00	Past Infection	M	EGY	1972	43
**542**	112.3	5.2440	0.0047	524.40	Past Infection	M	JOR	1964	51
**543**	99.3	4.4830	0.0045	448.30	Past Infection	M	SYR	1972	43
**544**	48.4	0.0000	0.0000	0.00	Past Infection	M	SYR	1974	41
**545**	83.3	1.3680	0.0016	136.80	Past Infection	M	NEP	1965	50
**546**	50.3	0.0000	0.0000	0.00	Past Infection	M	JOR	1954	61
**547**	40	1.3410	0.0034	134.10	Reactive Infection	M	ETH	1984	31
**548**	55.4	0.0000	0.0000	0.00	Past Infection	M	YEM	1985	30
**549**	289.2	0.0000	0.0000	0.00	Past Infection	M	QAT	1982	33
**550**	50.2	13.8490	0.0276	1384.90	Past Infection	M	PAL	1958	57
**551**	39.8	0.0000	0.0000	0.00	Past Infection	M	EGY	1981	34
**552**	55.8	55.4110	0.0993	5541.10	Reactive Infection	M	JOR	1974	41
**553**	50.4	2.7070	0.0054	270.70	Past Infection	M	SUD	1970	45
**554**	48.4	29.5000	0.0610	2950.00	Past Infection	F	PHI	1979	36
**555**	63.1	35.4120	0.0561	3541.20	Past Infection	M	QAT	1982	33
**556**	48.7	12.6510	0.0260	1265.10	Past Infection	M	PAL	1968	47
**557**	66.2	1.3140	0.0020	131.40	Past Infection	M	SYR	1979	36
**558**	56.4	4.8430	0.0086	484.30	Past Infection	M	EGY	1974	41
**559**	34.1	0.0000	0.0000	0.00	Past Infection	M	JOR	1986	29
**560**	39.1	0.0000	0.0000	0.00	Past Infection	M	SYR	1981	34
**561**	71.1	12.1180	0.0170	1211.80	Reactive Infection	M	EGY	1972	43
**562**	45.2	8.1300	0.0180	813.00	Past Infection	M	IND	1985	30
**563**	62.9	0.0000	0.0000	0.00	Past Infection	M	PAK	1973	42
**564**	65.2	3.6200	0.0056	362.00	Past Infection	M	QAT	1971	44
**565**	75.6	3.9030	0.0052	390.30	Past Infection	M	EGY	1979	36
**566**	79.5	0.0000	0.0000	0.00	Past Infection	M	EGY	1978	37
**567**	79.4	1.1400	0.0014	114.00	Past Infection	M	BAN	1966	49
**568**	119.3	0.0000	0.0000	0.00	Past Infection	M	EGY	1983	32

**569**	88	2.8000	0.0032	280.00	Past Infection	M	QAT	1971	44
**570**	113.6	2.2600	0.0020	226.00	Past Infection	M	EGY	1972	43
**571**	62.6	1.2300	0.0020	123.00	Recent Active	M	QAT	1992	23
**572**	82.5	18.2600	0.0221	1826.00	Past Infection	M	QAT	1973	42
**573**	64.5	0.0000	0.0000	0.00	Past Infection	M	PAL	1984	31
**574**	68.8	1.3360	0.0019	133.60	Past Infection	M	SYR	1989	26
**575**	56.3	15.0530	0.0267	1505.30	Past Infection	M	SYR	1989	26
**576**	53.4	0.0000	0.0000	0.00	Past Infection	M	EGY	1972	43
**577**	58.1	3.6000	0.0062	360.00	Past Infection	M	QAT	1953	62
**578**	58.6	0.0000	0.0000	0.00	Past Infection	M	EGY	1980	35
**579**	58.6	0.0000	0.0000	0.00	Past Infection	M	EGY	1981	34
**580**	49.2	1.1010	0.0022	110.10	Reactive Infection	M	UK	1955	60
**581**	91.5	1.2650	0.0014	126.50	Past Infection	M	SYR	1987	28
**582**	83.7	0.0000	0.0000	0.00	Past Infection	M	IND	1984	31
**583**	78.2	12.8200	0.0164	1282.00	Past Infection	M	SYR	1981	34
**584**	85.5	7.0990	0.0083	709.90	Past Infection	M	SYR	1978	37
**585**	147	0.0000	0.0000	0.00	Past Infection	M	IND	1991	24
**586**	73.9	71.1750	0.0963	7117.50	Past Infection	M	SYR	1976	39
**587**	98.7	2.8300	0.0029	283.00	Past Infection	M	IND	1981	34
**588**	102.2	0.0000	0.0000	0.00	Past Infection	M	IND	1985	30
**589**	111.8	6.6400	0.0059	664.00	Past Infection	M	MOR	1973	42
**591**	107	4.4890	0.0042	448.90	Past Infection	M	BAN	1981	34
**592**	83.8	1.2700	0.0015	127.00	Past Infection	M	EGY	1980	35
**593**	105.9	1.1810	0.0011	118.10	Past Infection	M	SYR	1988	27
**594**	107.9	1.3300	0.0012	133.00	Past Infection	M	QAT	1983	32
**595**	99.3	25.8000	0.0260	2580.00	Past Infection	M	EGY	1954	61
**596**	58.6	0.0000	0.0000	0.00	Past Infection	M	QAT	1995	20
**597**	56.1	1.2300	0.0022	123.00	Past Infection	M	IND	1989	26
**598**	60.4	0.0000	0.0000	0.00	Past Infection	M	IND	1983	32
**599**	109.1	1.3110	0.0012	131.10	Past Infection	M	QAT	1982	33

**600**	73.2	1.4900	0.0020	149.00	Past Infection	M	PAL	1982	33
**601**	104.2	12.6430	0.0121	1264.30	Past Infection	M	EGY	1985	30
**602**	59.4	0.0000	0.0000	0.00	No Infection	M	SYR	1977	38
**603**	74.1	2.2560	0.0030	225.60	Past Infection	M	PHI	1975	40
**604**	59.2	517.1000	0.8735	51710.00	Past Infection	M	QAT	1975	40
**605**	72.2	0.0000	0.0000	0.00	Past Infection	M	PAL	1959	56
**607**	111.3	33.8200	0.0304	3382.00	Past Infection	M	QAT	1975	40
**608**	103.8	0.0000	0.0000	0.00	Past Infection	M	EGY	1981	34
**609**	121.1	0.0000	0.0000	0.00	Past Infection	M	PAK	1976	39
**610**	63.5	0.0000	0.0000	0.00	Early Infection	F	MOR	1977	38
**611**	82.6	9.4830	0.0115	948.30	Past Infection	M	SYR	1982	33
**612**	109	0.0000	0.0000	0.00	Past Infection	M	BAN	1982	33
**613**	106	1.1300	0.0011	113.00	Past Infection	M	PHI	1969	46
**614**	67.1	2.3000	0.0034	230.00	Past Infection	M	SYR	1981	34
**615**	111.8	0.0000	0.0000	0.00	Past Infection	M	SOM	1993	22
**616**	73.6	0.0000	0.0000	0.00	Reactive Infection	M	QAT	1996	19
**617**	118.4	0.0000	0.0000	0.00	Past Infection	M	JOR	1979	36
**619**	84.8	0.0000	0.0000	0.00	Past Infection	M	QAT	1987	28
**620**	71	0.0000	0.0000	0.00	Past Infection	M	QAT	1973	42
**641**	74.9	9.5666	0.0128	956.66	Past Infection	M	QAT	1979	36
**642**	74.1	1.4972	0.0020	149.72	Past Infection	M	QAT	1977	38
**643**	73.4	2.2577	0.0031	225.77	Past Infection	M	QAT	1975	40
**644**	37.3	20.5161	0.0550	2051.61	Past Infection	M	QAT	1985	30
**645**	89.5	44.8723	0.0501	4487.23	Early Infection	M	QAT	1993	22
**646**	85.2	0.0000	0.0000	0.00	Past Infection	M	QAT	1981	34
**647**	68.9	0.0000	0.0000	0.00	Past Infection	M	QAT	1970	45
**648**	45.4	0.0000	0.0000	0.00	No Infection	M	QAT	1989	26
**649**	108.5	59.7680	0.0551	5976.80	Past Infection	M	QAT	1954	61
**650**	88.8	1.3560	0.0015	135.60	Past Infection	M	QAT	1962	53
**651**	38.7	14.2760	0.0369	1427.60	Past Infection	M	QAT	1963	52

**652**	61.7	0.0000	0.0000	0.00	Past Infection	M	QAT	1981	34
**653**	42.8	1.4182	0.0033	141.82	Past Infection	M	QAT	1959	56
**711**	40.5	0.0000	0.0000	0.00	Past Infection	M	QAT	1988	27
**712**	30.4	0.0000	0.0000	0.00	No Infection	M	QAT	1980	35
**713**	51.6	0.0000	0.0000	0.00	Past Infection	M	QAT	1979	36
**714**	90.9	0.0000	0.0000	0.00	Past Infection	M	QAT	1980	35
**715**	54.2	11.8141	0.0218	1181.41	Past Infection	M	QAT	1986	29
**716**	41.9	0.0000	0.0000	0.00	Past Infection	M	QAT	1990	25
**717**	68.5	54.3172	0.0793	5431.72	Past Infection	M	QAT	1983	32
**718**	66.9	0.0000	0.0000	0.00	Past Infection	M	QAT	1989	26
**719**	59.3	0.0000	0.0000	0.00	Past Infection	M	QAT	1989	26
**720**	39.9	10.7904	0.0270	1079.04	Past Infection	M	QAT	1973	42
**721**	65.5	0.0000	0.0000	0.00	Past Infection	M	QAT	1989	26
**722**	81.6	14.4965	0.0178	1449.65	Past Infection	M	QAT	1970	45
**723**	68.3	0.0000	0.0000	0.00	Past Infection	M	QAT	1982	33
**724**	45.1	0.0000	0.0000	0.00	Past Infection	M	QAT	1972	43
**725**	58	109.8341	0.1894	10983.41	Past Infection	M	QAT	1976	39
**726**	67.4	66.1056	0.0981	6610.56	Past Infection	M	QAT	1976	39
**727**	72.4	2.8310	0.0039	283.10	Reactive Infection	M	QAT	1984	31
**728**	65.1	0.0000	0.0000	0.00	Past Infection	M	QAT	1974	41
**752**	65.5	0.0000	0.0000	0.00	Past Infection	M	QAT	1974	41
**753**	86.2	3.8693	0.0045	386.93	Past Infection	M	QAT	1975	40
**754**	92.3	0.0000	0.0000	0.00	Past Infection	M	QAT	1991	24
**755**	116.6	0.0000	0.0000	0.00	Past Infection	M	QAT	1982	33
**756**	170.2	3.0025	0.0018	300.25	Past Infection	M	QAT	1978	37
**757**	87.7	3.7985	0.0043	379.85	Reactive Infection	M	QAT	1952	63
**758**	144.6	78.1298	0.0540	7812.98	Past Infection	M	QAT	1989	26
**759**	737.7	1.7477	0.0002	174.77	Past Infection	M	QAT	1996	19
**818**	90.4	84.9637	0.0940	8496.37	Past Infection	M	QAT	1959	56
**819**	87	6.1233	0.0070	612.33	Past Infection	M	QAT	1982	33

**893**	82.9	0.0000	0.0000	0.00	Past Infection	M	QAT	1992	23
**894**	95.8	0.0000	0.0000	0.00	Past Infection	M	QAT	1970	45
**895**	70.9	1.3520	0.0019	135.20	Past Infection	M	QAT	1974	41
**896**	94	0.0000	0.0000	0.00	Past Infection	M	QAT	1979	36
**897**	65.4	4.1190	0.0063	411.90	Past Infection	M	QAT	1981	34
**898**	52.7	1.1950	0.0023	119.50	Reactive Infection	M	QAT	1969	46
**899**	83.3	0.0000	0.0000	0.00	Past Infection	M	QAT	1982	33
**900**	52.5	12.9849	0.0247	1298.49	Past Infection	M	QAT	1979	36
**901**	91.9	2.6537	0.0029	265.37	Reactive Infection	F	QAT	1974	41
**902**	76.7	0.0000	0.0000	0.00	Reactive Infection	M	QAT	1988	27
**903**	137.5	33.8404	0.0246	3384.04	Past Infection	M	QAT	1969	46
**904**	120.7	2.5640	0.0021	256.40	Reactive Infection	M	QAT	1993	22
**918**	58.2	16.0455	0.0276	1604.55	Past Infection	M	QAT	1965	50
**919**	96.5	4.0851	0.0042	408.51	Past Infection	M	QAT	1964	51
**920**	32.3	1.5380	0.0048	153.80	Past Infection	M	QAT	1970	45
**921**	79.3	2.3263	0.0029	232.63	Past Infection	M	QAT	1984	31
**922**	67.1	1.4247	0.0021	142.47	Past Infection	M	QAT	1985	30
**923**	84.1	29.2655	0.0348	2926.55	Reactive Infection	M	QAT	1982	33
**924**	43.3	2.4548	0.0057	245.48	Reactive Infection	M	QAT	1971	44
**957**	35.6	0.0000	0.0000	0.00	Past Infection	M	QAT	1988	27
**958**	41.8	6.4357	0.0154	643.57	Past Infection	M	QAT	1986	29
**961**	37.9	2.3727	0.0063	237.27	Past Infection	M	QAT	1979	36
**962**	22.5	1.4463	0.0064	144.63	Past Infection	M	QAT	1991	24
**963**	29.7	2.7850	0.0094	278.50	Reactive Infection	M	QAT	1976	39
**964**	26.6	0.0000	0.0000	0.00	Past Infection	M	QAT	1958	57
**965**	45.1	7.5952	0.0168	759.52	Past Infection	M	QAT	1969	46
**966**	61.2	0.0000	0.0000	0.00	Past Infection	M	QAT	1987	28
**967**	31.5	1.4186	0.0045	141.86	Past Infection	M	QAT	1966	49
**968**	37.7	0.0000	0.0000	0.00	Past Infection	M	QAT	1967	48
**969**	33.1	1.3830	0.0042	138.30	Past Infection	M	QAT	1982	33

**970**	23.6	8.1771	0.0346	817.71	Past Infection	M	QAT	1985	30
**971**	58.1	65.3502	0.1125	6535.02	Past Infection	M	QAT	1974	41
**1010**	27.5	0.0000	0.0000	0.00	Past Infection	M	QAT	1964	51
**1011**	106.5	11.8913	0.0112	1189.13	Past Infection	M	QAT	1984	31
**1012**	54.8	6.5670	0.0120	656.70	Past Infection	M	QAT	1959	56
**1013**	41.1	0.0000	0.0000	0.00	Past Infection	M	QAT	1989	26
**1015**	32.3	12.6179	0.0391	1261.79	Past Infection	M	QAT	1979	36
**1016**	45.7	0.0000	0.0000	0.00	Past Infection	M	QAT	1972	43
**1017**	32.9	12.3957	0.0377	1239.57	Past Infection	M	QAT	1974	41
**1018**	38.2	0.9280	0.0024	92.80	Reactive Infection	M	QAT	1975	40
**1019**	30.6	0.0000	0.0000	0.00	Past Infection	M	QAT	1987	28
**1020**	36.6	0.0000	0.0000	0.00	Past Infection	M	QAT	1996	19
**1021**	58.4	99.8180	0.1709	9981.80	Past Infection	M	QAT	1969	46
**1057**	45.8	0.0000	0.0000	0.00	Past Infection	M	QAT	1971	44
**1058**	46.3	0.0000	0.0000	0.00	Past Infection	M	QAT	1985	30
**1059**	64.7	0.0000	0.0000	0.00	Past Infection	M	QAT	1978	37
**1060**	60.4	4.8660	0.0081	486.60	Past Infection	M	QAT	1970	45
**1061**	93.7	3.6160	0.0039	361.60	Past Infection	M	QAT	1968	47
**1099**	29.9	0.0000	0.0000	0.00	No Infection	M	QAT	1982	33
**1100**	46.1	34.5690	0.0750	3456.90	Past Infection	M	QAT	1982	33
**1112**	78.1	4.7410	0.0061	474.10	Past Infection	M	QAT	1974	41
**1113**	46	2.4040	0.0052	240.40	Past Infection	M	QAT	1976	39
**1114**	51.9	7.3060	0.0141	730.60	Past Infection	M	QAT	1969	46
**1115**	44.1	0.0000	0.0000	0.00	Past Infection	M	QAT	1975	40
**1116**	56.5	4.8660	0.0086	486.60	Past Infection	M	QAT	1977	38
**1117**	61.5	44.9550	0.0731	4495.50	Past Infection	M	QAT	1968	47
**1118**	47.6	0.0000	0.0000	0.00	Past Infection	M	QAT	1974	41
**1119**	48	0.0000	0.0000	0.00	Past Infection	M	QAT	1984	31
**1120**	29.6	0.0000	0.0000	0.00	Reactive Infection	M	QAT	1982	33
**1121**	37.7	0.7970	0.0021	79.70	Past Infection	M	QAT	1982	33

**1122**	25.3	17.8780	0.0707	1787.80	Past Infection	M	QAT	1950	65
**1123**	39.4	0.0000	0.0000	0.00	Past Infection	M	QAT	1982	33
**1124**	48.5	26.4260	0.0545	2642.60	Reactive Infection	M	QAT	1979	36
**1125**	37.6	0.0000	0.0000	0.00	Past Infection	M	QAT	1964	51
**1145**	32.4	0.0000	0.0000	0.00	Past Infection	M	QAT	1974	41
**1146**	73.1	9.6130	0.0132	961.30	Past Infection	M	QAT	1969	46
**1147**	21.2	0.0000	0.0000	0.00	Past Infection	M	QAT	1988	27
**1149**	39.6	9.9270	0.0251	992.70	Reactive Infection	M	QAT	1947	68
**1179**	63.9	3.4430	0.0054	344.30	Past Infection	M	QAT	1984	31
**1180**	43.4	0.6140	0.0014	61.40	Reactive Infection	M	QAT	1995	20
**1197**	55.9	0.0000	0.0000	0.00	No Infection	M	QAT	1996	19
**1249**	56.5	12.2210	0.0216	1222.10	Past Infection	M	QAT	1977	38
**1250**	52.5	6.3440	0.0121	634.40	Past Infection	M	QAT	1978	37
**1251**	29.2	1.1080	0.0038	110.80	Past Infection	M	QAT	1972	43
**1252**	31.3	3.5940	0.0115	359.40	Past Infection	M	QAT	1985	30
**1253**	47.7	3.7660	0.0079	376.60	Past Infection	M	QAT	1973	42
**1254**	49.8	0.0000	0.0000	0.00	Reactive Infection	M	QAT	1970	45
**1255**	43.3	6.4300	0.0148	643.00	Past Infection	M	QAT	1954	61
**1256**	37.7	0.0000	0.0000	0.00	Past Infection	M	QAT	1978	37
