# Supplementary figures and images for: Prevalence and molecular profiling of Epstein Barr virus (EBV) among healthy blood donors from different nationalities in Qatar
Source: PLoS One. 2017 Dec 11;12(12):e0189033. doi: 10.1371/journal.pone.0189033 (PMC5724864; doi:10.1371/journal.pone.0189033)

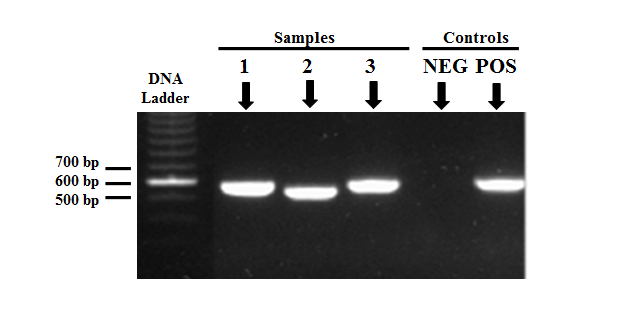

Supplement: S1 Fig — (TIF) [file pone.0189033.s002.tif]

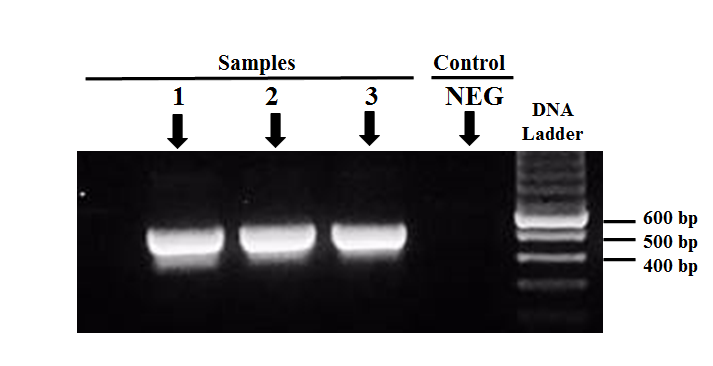

Supplement: S2 Fig — (TIF) [file pone.0189033.s003.tif]

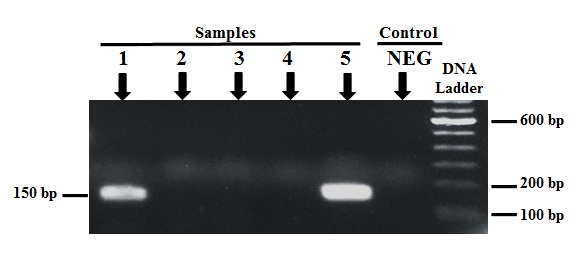

Supplement: S3 Fig — (TIF) [file pone.0189033.s004.tif]

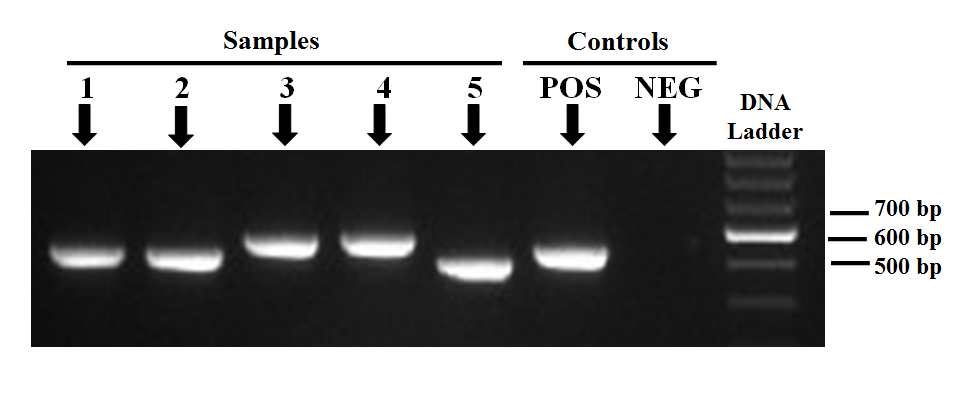

Supplement: S4 Fig — (TIF) [file pone.0189033.s005.tif]

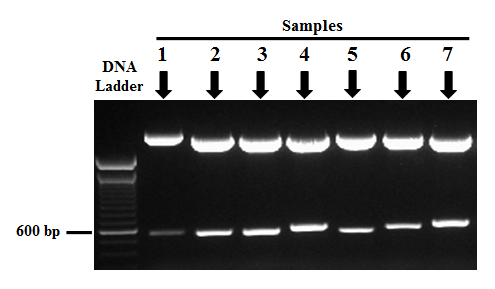

Supplement: S5 Fig — The 587 bp cloned fragments (Lane 1–7) were separated after digestion of the pDrive plasmid (upper thick band) with EcoRI. (TIF) [file pone.0189033.s006.tif]
